# Diagnosis of heart failure with preserved ejection fraction: a systematic narrative review of the evidence

**DOI:** 10.1007/s10741-023-10360-z

**Published:** 2023-10-20

**Authors:** Francesc Formiga, Julio Nuñez, María José Castillo Moraga, Marta Cobo Marcos, María Isabel Egocheaga, Concha F. García-Prieto, Angel Trueba-Sáiz, Arantxa Matalí Gilarranz, José María Fernández Rodriguez

**Affiliations:** 1 Servicio de Medicina Interna, Hospital Universitari de Bellvitge, Barcelona, Spain; 2 Servicio de Cardiología, Hospital Clínico Universitario de Valencia-España, Valencia, Spain; 3 Departamento de Medicina, Universidad de Valencia, Fundación de Investigación INCLIVA, Valencia, Spain; 4 Medicina Familiar y Comunitaria, Centro de Salud Barrio Bajo, Sanlúcar de Barrameda, Cádiz, Spain; 5 Servicio de Cardiología, Hospital Universitario Puerta de Hierro Majadahonda (IDIPHISA), Madrid, Spain; 6 Centro de Investigación Biomédica en Red en Enfermedades Cardiovasculares (CIBERCV), Madrid, Spain; 7 Medicina Familiar y Comunitaria, Centro de Salud Isla de Oza, Madrid, Spain; 8 Medical Affairs Department, Eli Lilly and Company España, Alcobendas, Madrid, Spain; 9 Medical Affairs Department, Boehringer Ingelheim, Sant Cugat del Vallés, Barcelona, Spain; 10 Área Cardiorrenometabólica del Servicio de Medicina Interna del Hospital Universitario Ramon y Cajal, Madrid, Spain; 11 Instituto Ramón y Cajal de Investigación Sanitaria (IRYCIS), Madrid, Spain

**Keywords:** Algorithm, Diagnosis, Diastolic function, Heart failure, Heart failure with preserved ejection fraction

## Abstract

Heart failure (HF) with preserved ejection fraction (HFpEF) is a common condition in clinical practice, affecting more than half of patients with HF. HFpEF is associated with morbidity and mortality and with considerable healthcare resource utilization and costs. Therefore, early diagnosis is crucial to facilitate prompt management, particularly initiation of sodium-glucose co-transporter 2 inhibitors. Although European guidelines define HFpEF as the presence of symptoms with or without signs of HF, left ventricular EF ≥ 50%, and objective evidence of cardiac structural and/or functional abnormalities, together with elevated natriuretic peptide levels, the diagnosis of HFpEF remains challenging. First, there is no clear consensus on how HFpEF should be defined. Furthermore, diagnostic tools, such as natriuretic peptide levels and resting echocardiogram findings, are significantly limited in the diagnosis of HFpEF. As a result, some patients are overdiagnosed (i.e., elderly people with comorbidities that mimic HF), although in other cases, HFpEF is overlooked. In this manuscript, we perform a systematic narrative review of the diagnostic approach to patients with HFpEF. We also propose a comprehensible algorithm that can be easily applied in daily clinical practice and could prove useful for confirming or ruling out a diagnosis of HFpEF.

**Supplementary Information:**

The online version contains supplementary material available at 10.1007/s10741-023-10360-z.

##  Introduction

Heart failure (HF) with preserved ejection fraction (HFpEF) is a common condition, affecting more than half of patients with HF [1, 2]. Moreover, with aging of the population, the prevalence of HFpEF is expected to increase in the coming years [2]. HFpEF is associated with high morbidity and mortality. A recent study showed that among patients with HFpEF, event rates for hospitalization with HF reach 198 per 1000 person-years [1]. In addition, healthcare resource utilization and costs among patients with HFpEF are huge [3]. Therefore, early diagnosis of HFpEF can play a key role in facilitating prompt initiation of drugs that reduce the burden of HF in this population [4, 5].

European guidelines define HFpEF as the presence of symptoms with or without signs of HF, left ventricular ejection fraction (LVEF) ≥ 50%, and objective evidence of cardiac structural and/or functional abnormalities (presence of left ventricular diastolic dysfunction/raised left ventricular filling pressure), including elevated natriuretic peptide levels [6]. However, the diagnosis of HFpEF remains challenging. First, there is no clear consensus on how HFpEF should be defined, including the LVEF cut-off to use [7]. In addition, whereas some authors consider that in many patients, particularly elderly people, HF is unrecognized [7], others state that HFpEF is overdiagnosed, as various conditions share symptoms and signs, mainly in patients with many comorbidities [8]. Furthermore, diagnostic tools, such as natriuretic peptide testing and resting echocardiogram, are subject to limitations in the diagnosis of HFpEF [9] or are not applied in many patients [10].

Therefore, with the aim of ensuring a more correct diagnosis of this syndrome, a group of multidisciplinary experts met to provide a simple and practical approach to the diagnosis of HFpEF based on a systematic narrative review of currently available evidence.

## Search strategy

A bibliographic search on the diagnostic approach to HFpEF was performed using MEDLINE and Embase. The strategy was carried out using the OVID meta-search engine in the case of Embase. The search was performed on December 27, 2022, and included references from 2016 to that date. References in English and Spanish were included.

Two strategies were applied for the different databases with the keywords of interest, namely both the MeSH/Emtree terms from the PubMed/Embase thesaurus and the free text terminological variants in the title or in the abstract. Standard date filters from 2016 and a language filter (English and Spanish) were applied. As the most recent articles in the database did not have MeSH/Emtree terms assigned, respectively, in the PubMed/Embase databases, which were consulted through the OVID metasearch engine, specific strategies were created using the full-text search in the title and/or summary and included HF (heart failure), LVEF (left ventricular ejection fraction), and diagnostic tools. The initial search strategies recorded 134 references in Embase (OVID) and 280 references in MEDLINE (PubMed) (Supplementary material). After eliminating duplicate references with the reference management software (Zotero 6.0), a total of 377 references were recovered. These were subsequently reduced to 185 after manual selection (PRISMA flow chart is shown in supplementary Fig. 2).

## Diagnosis of HF

HF is a clinical syndrome with symptoms and/or signs caused by structural and/or functional cardiac abnormalities and confirmed by elevated natriuretic peptide levels and/or objective evidence of pulmonary or systemic congestion [11]. Therefore, these aspects should be taken into consideration when attempting to confirm a diagnosis of HFpEF.

### Clinical suspicion

Diagnosis of HF requires the presence of symptoms with or without signs of HF. Typical symptoms include breathlessness, orthopnea, paroxysmal nocturnal dyspnea, reduced exercise tolerance, fatigue, and ankle swelling; typical signs include peripheral edema, lung rales, elevated jugular venous pressure, or third heart sound [6]. However, since symptoms and signs alone are not sufficiently accurate to confirm a diagnosis of HF, additional diagnostic tools are required to correctly diagnose HFpEF [12, 13].

Dyspnea is the cardinal symptom of HFpEF. However, patients with HFpEF are usually elderly and may have many comorbidities, some of which can mimic HF, such as coronary artery disease, lung disease, obesity, diabetes, atrial fibrillation, and anemia. Therefore, these comorbidities should be ruled out, or, at least, their contribution to symptomatology should be determined [14, 15]. Importantly, it is mandatory to investigate whether dyspnea has a respiratory or a cardiac origin. Table 1 shows key aspects that may be helpful to differentiate between them [16].

**Table 1:** Dyspnea: pulmonary vs cardiac origin

	**Respiratory**	**Chronic heart failure**
**Clinical course**	Long and recurrent	Progressive
**Physical examination**	Snoring and wheezing Muffled heart tones	Crackles Murmur, S3, S4
**Chest X-ray**	Normal heart size Interstitial pattern Pulmonary hypertension	Cardiomegaly Interstitial/alveolar edema Venous-capillary hypertension
**Electrocardiogram**	Normal, right ventricular overload, low voltage, right bundle branch block, atrial fibrillation/flutter	Abnormal: left ventricular hypertrophy, ST-T alterations, Q waves, left bundle branch block, atrial fibrillation/flutter
**Respiratory function**	Obstruction	Normal or mild restriction
**Response to diuretics**	−	++
**Response to bronchodilators**	++	−

Table based on data from reference #16

Another key point is that no symptoms or signs by themselves can help us to determine whether a patient has HFpEF or HF with reduced EF. For example, the CHARM program included three clinical trials, two of which enrolled patients with HF with reduced EF and one with patients with HFpEF. Although some symptoms or signs could be more common in one type of HF than the other, they are frequent in both HFpEF and HF with reduced EF (Table 2) [17].

**Table 2:** Symptoms and signs of heart failure with preserved ejection fraction vs heart failure with reduced ejection fraction

	**HFpEF (CHARM preserved)**	**HFrEF (CHARM added and alternative)**
Edema, %	30	23–25
Orthopnea, %	19	20–21
Cardiomegaly, %	16	25–26
Basal crackles, %	15	15–16
Paroxysmal nocturnal dyspnea, %	12	13–14
Dyspnea at rest, %	10	12–13
Jugular venous pressure > 6 cm, %	7	9–10
Third heart sound, %	5	16–18
Upper-zone redistribution, %	2	3

Table based on data from reference #17

*HFpEF* heart failure with preserved ejection fraction, *HFrE*F heart failure with reduced ejection fraction

### Electrocardiogram, chest X-ray, and lung ultrasound

The evaluation of patients with suspected HF should include an electrocardiogram, as a normal electrocardiogram is unusual in patients with HF. In addition, it may be helpful to consider the etiology of HF (arterial hypertension [left ventricular hypertrophy, systolic overload]; ischemic heart disease [ST-T alterations, Q waves]). Electrocardiogram abnormalities, such as atrial fibrillation/flutter, conduction disorders, left ventricular hypertrophy, pathologic Q waves, ST-T segment alterations, and left bundle branch block, are common in patients with HF [6, 18].

A chest X-ray may provide supportive evidence of HF, such as pulmonary congestion or cardiomegaly, although it can also be used to investigate other potential causes of dyspnea, particularly pulmonary diseases [6]. Moreover, the use of lung ultrasound can help in the diagnosis of HF and may have prognostic value, as the number of B-lines is associated with adverse outcomes [19].

### Natriuretic peptides

European guidelines recommend determination of natriuretic peptide levels to rule out the diagnosis in patients with symptoms suggestive of HF [6]. However, natriuretic peptide levels are increased not only in HF but also in other clinical conditions (acute setting [acute coronary syndrome, atrial or ventricular arrhythmias, pulmonary embolism, acute kidney disease, sepsis]; chronic setting [increasing age, chronic kidney disease, left ventricular hypertrophy, chronic obstructive pulmonary disease, atrial fibrillation]) [6, 20]. By contrast, natriuretic peptide levels may be disproportionately low in obese patients. In fact, low NT-proBNP levels in overweight and obese patients do not rule out the diagnosis of HFpEF [21]. In this context, European guidelines recommend an upper limit of normal in the non-acute setting of 35 pg/mL for BNP and 125 pg/mL for NT-proBNP, as these values have a very high negative predictive value (from 0.94 to 0.98) and values under these levels make a diagnosis of HF very unlikely [6]. However, it should be noted that, for the same NYHA functional class, natriuretic peptide levels are higher in patients with HF with reduced EF than in patients with HFpEF and that in patients with HFpEF in NYHA functional class I or II, natriuretic peptide levels are not markedly increased [22]. In addition, many conditions that may modify natriuretic peptide levels are also common in patients with HFpEF [6]. Therefore, higher natriuretic peptide levels should be considered to rule out a diagnosis of HFpEF in this population. In fact, recent clinical trials enrolling patients with HFpEF (i.e., EMPEROR-Preserved [NT-proBNP: sinus rhythm: > 300 pg/mL; atrial fibrillation; > 900 pg/mL], DELIVER [NT-proBNP: sinus rhythm: ≥ 300 pg/mL; atrial fibrillation; ≥ 600 pg/mL], and PARAGON-HF [NT-proBNP: sinus rhythm: > 300 pg/mL; atrial fibrillation; > 900 pg/mL]) have defined higher cut-off levels of NT-proBNP as inclusion criteria (Table 3) [4, 5, 23]. As a result, we recommend as cut-off levels for NT-proBNP ≥ 300 pg/mL if sinus rhythm and ≥ 600 pg/mL if atrial fibrillation. In patients with low natriuretic peptide levels in whom HFpEF is suspected, the risk of adverse outcomes is much lower [24]. In this context, the HFA-PEFF score proposes higher levels of natriuretic peptides for a diagnosis of HFpEF (Table 4) [25–28]. On the other hand, as BNP seems a worse marker than NT-proBNP for the diagnosis of HFpEF, the latter would be better in this clinical setting [29]. Finally, other biomarkers tested in HFpEF include high-sensitivity troponins and novel biomarkers, particularly soluble suppression of tumorigenesis-2, galectin-3 (Gal-3), growth differentiation factor 15 (GDF-15), and carbohydrate antigen 125 (CA125), which have been shown to predict adverse outcomes independently from natriuretic peptide levels, as well as EF [30–32]. Biomarkers such as soluble glycoprotein 130 and heat shock protein 27 (hsp27) have also been proposed as biomarkers of chronic HFpEF [33].

**Table 3:** Cut-off levels for natriuretic peptides in the diagnosis of HFpEF in the 2021 HF ESC guidelines and in the EMPEROR-preserved, DELIVER, and PARAGON HF trials

	**2021 HF ESC guidelines**	**EMPEROR- Preserved**	**DELIVER**	**PARAGON-HF**
**NT-proBNP, pg/mL**				
Sinus rhythm	≥ 125	> 300	≥ 300	> 300; if HFh within 9 months > 200
Atrial fibrillation	≥ 125	> 900	≥ 600	> 900; if HFh within 9 months > 600
**BNP, pg/mL**				
Sinus rhythm	≥ 35	–	–	–
Atrial fibrillation	≥ 35	–	–	–

Table based on data from references #4–6, 23

*ESC* European Society of Cardiology, *HFpEF* heart failure with preserved ejection fraction, *HFh* heart failure hospitalization

**Table 4:** Scoring algorithms for diagnosis of heart failure with preserved ejection fraction

**H2FPEF**			**HFA-PEFF**			
H_2_	**H**eavy (BMI > 30 kg/m^2^)	2 points	Functional echocardiographic parameters			
	**H**ypertension (≥ 2 drugs)	1 point	Septal e′ < 7 cm/s Lateral e′ < 10 cm/sec Average E/e′ ≥ 15 Tricuspid regurgitation velocity > 2.8 m/s	2 points	Average E/e′ ≥ 9–14 Global longitudinal strain < 16%	1 point
F	Atrial **f**ibrillation	3 points	Morphological echocardiographic parameters			
P	**P**ulmonary hypertension (pulmonary artery systolic pressure > 35 mmHg)	1 point	Left atrium volume index > 34 mL/m^2^ Left ventricular mass index ≥ 149/122 g/m^2^ (male/female) and relative wall thickness > 0.42	2 points	Left atrium volume index 29–34 mL/m^2^ Left ventricular mass index > 115/95 g/m^2^ (male/female) Relative wall thickness > 0.42 Left ventricular wall thickness ≥ 12 mm	1 point
E	**E**lder (age > 60 years)	1 point	Biomarkers			
F	Elevated **f**illing pressure (E/e > 9 by echocardiogram)	1 point	Sinus rhythm: • NT-proBNP: > 220 pg/mL • BNP: > 80 pg/mL Atrial fibrillation • NT-proBNP: > 660 pg/mL • BNP: > 240 pg/mL	2 points	Sinus rhythm: • NT-proBNP: 150–220 pg/mL • BNP: 35–80 pg/mL Atrial fibrillation • NT-proBNP: 365–660 pg/mL • BNP: 105–240 pg/mL	1 point
Score: - 0–1 points: low probability of HFpEF - 2–5 points: intermediate probability of HFpEF - 6–9 points: high probability of HFpEF			Score: - 2–4 points: intermediate probability of HFpEF - ≥ 5 points: high probability of HFpEF			

Table based on data from references #25, 26

*BMI* body mass index, *HFpEF* heart failure with preserved ejection fraction

### Echocardiography

Echocardiography is the key diagnostic tool in HFpEF. It provides relevant information about functional and morphological aspects of the heart [6, 34]. Thus, the echocardiogram enables us to determine the left ventricular and right ventricular ejection fraction, chamber size, and valvular function, as well as the presence of regional wall motion abnormalities, eccentric and concentric left ventricular hypertrophy, pulmonary hypertension, and markers of diastolic function [6, 26].

The echocardiographic workflow should be standardized [34]. The first step in identifying HFpEF is determination of LVEF, which should be measured, rather than estimated, ideally from biplane or three-dimensional images. Left ventricular diameters and volumes should then be recorded, with a special focus on assessing the presence of concentric remodeling or left ventricular hypertrophy and non-dilated left ventricle and left atrial enlargement [26, 34]. Although the presence of concentric left ventricular remodeling or hypertrophy renders a diagnosis of HFpEF more likely, its absence does not necessarily exclude the diagnosis of HFpEF. On the other hand, after excluding valvular heart disease, left atrial enlargement reflects chronically elevated left ventricular filling pressure (with or without atrial fibrillation) [6, 26].

The next stage should involve estimation of left ventricular filling pressure or pulmonary capillary wedge pressure using transthoracic echocardiography. These parameters include early (E) and late diastolic mitral inflow velocity (mitral E/A ratio), septal and lateral mitral annular early diastolic velocity (e′), ratio of early diastolic mitral inflow and annular velocity (E/e′ ratio), maximal left atrial volume index, and tricuspid regurgitation peak velocity, which enables measurement of pulmonary artery systolic pressure [35, 36]. The E/e′ ratio is usually considered the first step when assessing diastolic function. A mean E/e′ index ≥ 15 at rest identifies patients with high mean pulmonary capillary wedge pressure, thus making a diagnosis of HFpEF more likely. However, a value of 9–14 is less sensitive and should be considered a minor criterion. As E/e′ is subject to limitations [37, 38], this parameter should not be considered alone and should be included within a comprehensive echocardiographic approach for the diagnosis of HFpEF. A recent study showed that a multivariable-based approach including different parameters assessed using echocardiography increases accuracy in the diagnosis of HFpEF. In other words, the greater the number of echocardiographic abnormalities, the higher the likelihood of HFpEF [39]. The structural and functional alterations for the diagnosis of HFpEF using echocardiography are summarized in Table 5 [6, 26, 40, 41].

**Table 5:** Structural and functional alterations for the diagnosis of HFpEF by echocardiography

**2021 HF ESC guidelines**		**2019 HFA-ESC consensus**		
**Functional or structural criteria**			**Functional**	**Structural**
LV mass index	≥ 95/115 g/m^2^ (female/male)	**Major criteria**	Septal e′ < 7 cm/sec or Lateral e′ < 10 cm/sec or Average E/e′ ≥ 15 or Tricuspid regurgitation velocity > 2.8 m/s (PA systolic pressure > 35 mmHg)	LA volume index > 34 ml/m^2^ or LV mass index ≥ 149/122 g/m^2^ (male/female) and relative wall thickness > 0.42
Relative wall thickness	> 0.42			
LA volume index	> 34 mL/m^2^ (SR) > 40 mL/m^2^ (AF)			
E/e′ratio at rest	> 9	**Minor criteria**	Average E/e′ ≥ 9–14 or Global longitudinal strain < 16%	LA volume index 29–34 ml/m^2^ or LV mass index > 115/95 g/m^2^ (male/female) or Relative wall thickness > 0.42 or Left ventricular wall thickness ≥ 12 mm
PA systolic pressure	> 35 mmHg			
TR velocity at rest	> 2.8 m/s			
**Oh et al.**				
e′ ≤ 6 cm/s or MAC* and E/A ≥ 1.5 e′ ≤ 6 cm/s or MAC*, E/A 0.8– < 1.5, and tricuspid regurgitation ≥ 2.8 m/s or LA reservoir strain ≤ 24% e′ 6– < 9 cm/s or AF and E/e′ ≥ 15 e′ 6– < 9 cm/s or AF and E/e′ 9– < 15 and tricuspid regurgitation ≥ 2.8 m/s or LA reservoir strain ≤ 24%				Diagnosis of HFpEF
e′ ≤ 6 cm/s or MAC*, E/A 0.8– < 1.5 and tricuspid regurgitation < 2.8 m/s or LA reservoir strain > 24%: normal filling pressure e′ ≤ 6 cm/s or MAC*, E/A < 0.8: normal filling pressure e′ 6– < 9 cm/s or AF and E/A < 0.8: normal filling pressure e′ 6– < 9 cm/s or AF and E/e′ 9– < 15 and tricuspid regurgitation < 2.8 m/s or LA reservoir strain > 24%: normal filling pressure e′ 6– < 9 cm/s or AF and E/e′ < 9: normal filling pressure				Exercise diastolic echo or exercise catheterization
e′ ≥ 9 cm/s and SR				No HFpEF

Table based on data from references #6, 26, 41. Early to late diastolic transmitral flow velocity (E/A) ratio and the E to early diastolic mitral annular tissue velocity (E/e′) ratio

*AF* atrial fibrillation, *E/e′ ratio* early filling velocity on transmitral Doppler/early relaxation velocity on tissue Doppler, *ESC* European Society of Cardiology, *HFA* Heart Failure Association, *HFpEF* heart failure with preserved ejection fraction, *LA* left atrial, *LV* left ventricular, *MAC* mitral annulus calcification, *PA* pulmonary artery, *SR* sinus rhythm, *TR* tricuspid regurgitation

*MAC: in these patients, velocity is not reliable

It should be noted that, in some cases, access to a rapid echocardiography examination is difficult. Better coordination between healthcare levels is mandatory if we are to improve the diagnostic approach to patients with suspicion of HFpEF [26, 35]. In this context, the development of artificial intelligence–assisted echocardiography of HFpEF has been shown to be an accurate prescreening method capable of automatically generating quantitative metrics that could prove very valuable for clinicians [42].

Additional imaging techniques, such as cardiac magnetic resonance, can prove useful in cases of a doubtful diagnosis of HFpEF or when a particular etiology is suspected. In fact, cardiac magnetic resonance imaging provides relevant measurements for cardiac structure and function, enables tissue characterization, and could facilitate the early diagnosis of HFpEF. The main problem is its availability in daily clinical practice [43, 44].

### Scores

In this context, two scoring systems have been proposed to simplify the diagnostic approach to patients with HFpEF, namely H2FPEF and HFA-PEFF. While the H2FPEF score relies mostly on comorbidities, the HFA-PEFF scoring system is based on echocardiographic structural and functional parameters and natriuretic peptide levels (Table 4) [25, 26]. Different studies have analyzed the validity of these scores for the diagnosis of HFpEF in real-world practice. Although most have shown that they are reliable diagnostic tools in HFpEF, with high diagnostic accuracy, and are associated with diastolic dysfunction, lower cardiac output, and exercise intolerance, they are barely used in clinical practice and their results may be discordant in patients affected by unexplained dyspnea, with relevant differences in sensitivity and specificity according to the clinical setting [45–50].

### Additional diagnostic tools

Although the diagnosis of HFpEF can be reasonably performed in most patients after a clinical history, physical examination, measurement of biological parameters, and echocardiography, additional confirmatory tests may be needed when the diagnosis is not clear. In these cases, further investigation is required.

#### Diastolic stress test

Diastolic stress tests, mainly exercise echocardiography, can unmask left ventricular diastolic and systolic dysfunction and should be the next step when attempting to confirm a diagnosis of HFpEF. Of note, this is a mainly submaximal exercise stress test, whereas the maximal exercise stress test is generally used to exclude ischemia [6, 26, 51–54].

The parameters most commonly analyzed to rule out HFpEF are mitral E/e′ ratio and tricuspid regurgitation peak velocity, which are closely associated with mean pulmonary capillary wedge pressure and pulmonary artery systolic pressure, respectively. These parameters should be measured during standardized exercise. In addition, stroke volume and its change during exercise should also be determined. An average E/e′ ratio at peak stress ≥ 15 and a tricuspid regurgitation velocity > 3.4 m/s increase the probability of a diagnosis of HFpEF. In fact, an average E/e′ ratio at peak stress ≥ 15 adds two points to the HFA–PEFF score and three points when the two conditions are present. Additionally, the absence of increased cardiac output during exercise also favors HFpEF as the etiology of dyspnea [6, 26, 51–54].

#### Right heart catheterization

If exercise echocardiography cannot be performed or data are inconclusive, an invasive hemodynamic test is recommended. If the patient has an invasively measured pulmonary capillary wedge pressure of ≥ 15 mmHg or left ventricular end-diastolic pressure ≥ 16 mmHg at rest, then a diagnosis of HFpEF can be considered. If not, an invasive hemodynamic measurement of pulmonary capillary wedge pressure should be taken during exercise. In the case of pulmonary capillary wedge pressure ≥ 25 mmHg, the patient has HFpEF; if not, HFpEF can be ruled out [6, 26, 55]. It is important to note that this diagnostic procedure is subject to risks and may not always be available. In addition, invasive exercise hemodynamics is limited for the diagnosis of HFpEF, for example, it is subject to respiratory pressure swings that may impact on the results in up to 30% of patients [55]. Therefore, it should be limited to specific cases, particularly when therapy depends on the results [6, 26, 56].

Additionally, although further studies are required, the use of specific microRNA panels could add value to current biomarkers in the diagnosis of HFpEF [57].

## Etiology of HFpEF

Once the diagnosis of HFpEF has been confirmed, the underlying cause should be determined in order to initiate specific treatment. In most cases, HFpEF is associated with risk factors and comorbidities, particularly with long-term poorly controlled arterial hypertension. However, conditions that mimic HFpEF should be ruled out, for example, hypertrophic cardiomyopathy, inflammatory or infiltrative cardiomyopathy, and storage disease [6, 26]. Additionally, myocardial ischemia, an abnormal blood pressure response to exercise, chronotropic incompetence, and supraventricular and ventricular arrhythmias should also be investigated if the patient has clinical findings that suggest a history of any of these conditions. Therefore, specific diagnostic tools should be indicated according to the clinical suspicion (Table 6) [6, 26].

**Table 6:** Potential etiologies of HFpEF and specific diagnostic tests

**Etiology**	**Diagnostic tests**
Ischemic heart disease	CMR
Cardiomyopathy	CMR
Storage diseases	CMR, cardiac or non-cardiac biopsies
Myocarditis	CMR, cardiac or non-cardiac biopsies
Sarcoidosis	CMR, eosinophilia, IL-2 receptor, ACE, FDG/PET, PET/CT
Hemochromatosis	CMR, serum ferritin, genetic testing
Amyloidosis	Genetic testing (hATTR), Bence-Jones proteinuria (AL), 99mTc-DPD scintigraphy (transthyretin), global longitudinal strain with apical sparing, FDG/PET; PET/CT
Hypertrophic cardiomyopathy	Genetic testing
Restrictive cardiomyopathy	Genetic testing
Fabry disease	Alpha-galactosidase activity in leukocytes
Loeffler endomyocarditis	Eosinophilia
Constrictive pericarditis	CT

Table based on data from references #6, 26

*ACE* angiotensin-converting enzyme, *AL* amyloid light chain, *(h)ATTR* (hereditary) transthyretin-mediated amyloidosis, *CMR* cardiac magnetic resonance, *CT* computed tomography, *HFpEF* heart failure with preserved ejection fraction, *IL-2* interleukin 2, *FDG PET* positron emission tomography with 18f-fluorodeoxyglucose

In this clinical setting, it is important to exclude cardiac amyloidosis, which should be suspected in patients aged > 65 years with HF and left ventricular hypertrophy (septum ≥ 12 mm). Other parameters that increase the probability of cardiac amyloidosis include hypotension, which is more common in affected patients. Pseudo-infarct electrocardiographic pattern, low QRS voltage, and conduction abnormalities are typical findings on the electrocardiogram. Moreover, affected patients also have disproportionally elevated natriuretic peptide levels. In addition, granular sparkling of the myocardium, increased right ventricular wall thickness, pericardial effusion, and altered longitudinal strain can also be observed in echocardiography [6, 26, 58–61].

## Diagnostic algorithm

**Fig. 1:**
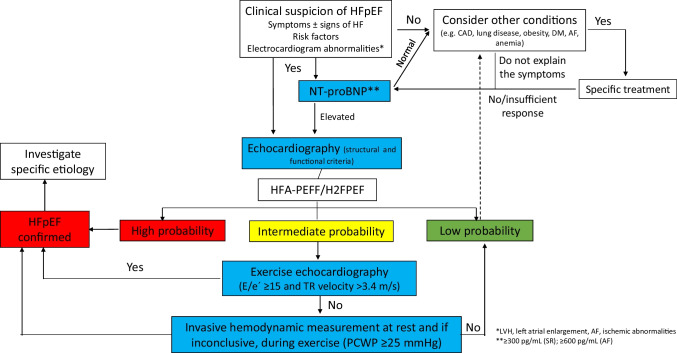
Diagnostic algorithm for HFpEF*. AF, atrial fibrillation; CAD, coronary artery disease; DM, diabetes mellitus; HFpEF, heart failure with preserved ejection fraction; LVH, left ventricular hypertrophy; PCWP, pulmonary capillary wedge pressure; SR, sinus rhythm; TR, tricuspid regurgitation. *The presence of HFpEF mimics (i.e., cardiac amyloidosis, hypertrophic cardiomyopathy, cardiac sarcoidosis, hemochromatosis, Fabry disease, high-output HF, myocarditis, pericardial disease) should be considered and ruled out when clinical suspicion exists. Figure based on data from references #6, 26, 41, 62–68

A diagnostic algorithm that can be translated into clinical practice is proposed in Fig. 1 [6, 26, 40, 41, 62–68]. The first step is clinical suspicion of HF. Not only should the symptoms and signs of HF be considered but also the presence of other comorbidities should also be taken into account when attempting to identify other causes of dyspnea or the contribution of these conditions to the patient’s clinical status. An electrocardiogram should then be performed, and natriuretic peptide levels (NT-proBNP) should be determined. We used the cut-off levels of clinical trials rather than ESC guidelines, to increase specificity. If any of the values are altered, echocardiography is mandatory (see criteria for HFpEF in Table 5). If all the data indicate a high probability, the diagnosis of HFpEF can be confirmed and further investigations can be considered if a specific etiology is suspected. Personalized treatment of HFpEF should be started early based on comorbidities and congestion status, with priority accorded to drugs showing an established clinical benefit, such as sodium-glucose co-transporter 2 inhibitors [4, 5]. If the probability of HFpEF is low, other cardiac and extracardiac causes of dyspnea should be considered. In the case of intermediate probability, invasive measurements can be performed to unmask left ventricular diastolic dysfunction [6, 26, 40, 41, 62–68]. On the other hand, there are some conditions (i.e., cardiac amyloidosis, hypertrophic cardiomyopathy, cardiac sarcoidosis, hemochromatosis, Fabry disease, high-output HF, myocarditis, pericardial disease) that in some cases can be considered as HFpEF mimics. As a result, these conditions should be taken into account and ruled out when clinical suspicion exists [68].

In recent years, several algorithms have been published regarding the diagnosis of HFpEF. Some of them are too complex, with a lot of information, which decreases their applicability in clinical practice. Others, however, are too simple and not all the necessary information to perform an accurate diagnosis is included or is not updated. That is why we think that our algorithm provides all the necessary information, without being too complex, and thus may be helpful to make an appropriate diagnostic approach for patients with suspected HFpEF. As a result, this is a comprehensible algorithm that should be implemented in clinical practice at different healthcare levels, including cardiology, internal medicine, and primary care. Such an approach will most likely increase awareness of the need for early identification of this entity and facilitate early diagnosis and the initiation of drugs with proven efficacy in the affected population.

## Conclusions

HFpEF is a very common condition that is associated with high morbidity and mortality. However, confirming a diagnosis of HFpEF is challenging, as affected patients have many comorbidities that can mimic the condition. Additionally, HFpEF is not defined based on a single criterion but on a cluster of parameters, mainly increased natriuretic peptide levels and specific echocardiographic alterations. We present a comprehensible algorithm that can easily be applied to real-world patients and prove useful when confirming or ruling out a diagnosis of HFpEF.

### Supplementary Information

Below is the link to the electronic supplementary material.

Supplementary file1 (DOCX 135 KB)

## Data Availability

Data sharing is not applicable to this article as no datasets were generated or analyzed during the current study.

## Declarations

### Medical writing/editorial assistance

Writing and editorial assistance was provided by Content Ed Net (Madrid, Spain), which was contracted and funded by Boehringer Ingelheim.
