# Correction to: Diagnosis of heart failure with preserved ejection fraction: a systematic narrative review of the evidence

**DOI:** 10.1007/s10741-023-10365-8

**Published:** 2023-11-13

**Authors:** Francesc Formiga, Julio Nuñez, María José Castillo Moraga, Marta Cobo Marcos, María Isabel Egocheaga, Concha F. García‑Prieto, Angel Trueba‑Sáiz, Arantxa Matalí Gilarranz, José María Fernández Rodriguez

**Affiliations:** 1 Servicio de Medicina Interna, Hospital Universitari de Bellvitge, Barcelona, Spain; 2 Servicio de Cardiología, Hospital Clínico Universitario de Valencia-España, Valencia, Spain; 3 Departamento de Medicina, Universidad de Valencia, Fundación de Investigación INCLIVA, Valencia, Spain; 4 Medicina Familiar y Comunitaria, Centro de Salud Barrio Bajo, Sanlúcar de Barrameda, Cádiz, Spain; 5 Servicio de Cardiología, Hospital Universitario Puerta de Hierro Majadahonda (IDIPHISA), Madrid, Spain; 6 Centro de Investigación Biomédica en Red en Enfermedades Cardiovasculares (CIBERCV), Madrid, Spain; 7 Medicina Familiar y Comunitaria, Centro de Salud Isla de Oza, Madrid, Spain; 8 Medical Affairs Department, Eli Lilly and Company España, Alcobendas, Madrid, Spain; 9 Medical Affairs Department, Sant Cugat del Vallés, Boehringer Ingelheim, Barcelona, Spain; 10 Área Cardiorrenometabólica del Servicio de Medicina Interna del Hospital Universitario Ramon y Cajal, Madrid, Spain; 11 Instituto Ramón y Cajal de Investigación Sanitaria (IRYCIS), Madrid, Spain

**Correction to: Heart Failure Reviews** **https://doi.org/10.1007/s10741-023-10360-z**

An oversight has occurred in the published version of our paper. Upon review, we have discovered that Fig. 1 did not match the updated version submitted for publication.

We sincerely apologize for any confusion this may have caused.

The original article has been corrected to reflect the appropriate and accurate figure.

