# Supplementary material for: Diagnosis of heart failure with preserved ejection fraction: a systematic narrative review of the evidence
Source: Heart Fail Rev. 2023 Oct 20;29(1):179–89. doi: 10.1007/s10741-023-10360-z (PMC10904432; doi:10.1007/s10741-023-10360-z)

**Supplementary material**

**1. Bibliographic search strategies**

**1.1.** [**EMBASE (OVID) - [134 items]**](#_Toc125645451)

1 exp heart failure/ (602974)

2 ((heart or cardia* or myocardial*) adj3 (failure or insufficienc* or descompensat*)).ti,ab.(359126)

3 1 or 2 (668135)

4 preserved ejection fraction.ab,ti. (13083)

5 preserved.ti,ab. adj3 (ejection fraction or ef or lvef).ab,ti. (19265)

6 HFpEF.ab,ti. (9391)

7 HF-pEF.ab,ti. (309)

8 4 or 5 or 6 or 7 (20555)

9 (left ventricle adj2 ejection fraction).ab,ti. (3507)

10 Ventricular Dysfunction/ or Ventricular Function/ (20896)

11 preserved.ti,ab. (167486)

12 (9 or 10) and 11 (1588)

13 (8 or 12) and 3 (17810)

14 (diagnosable or diagnosi or diagnosis or diagnose or diagnoses or diagnosed or diagnosing or theranostic).ab,ti. (3518790)

15 *diagnosis/ (62075)

16 (“decision making” or criteria* or tool* or algorithm*).ab,ti. (2837321)

17 (14 or 15) adj4 16 (92429)

18 13 and 17 (190)

19 limit 18 to yr=”2016 –Current” (145)

20 limit 19 to (english or spanish) (134)

1.2. [MEDLINE (PUBMED) - [280 items]](#_Toc125645452)

#1 "Heart Failure"[Mesh] 143,010

#2 (heart[Title/Abstract] OR cardia*[Title/Abstract] OR myocardial*[Title/Abstract]) AND (failure[Title/Abstract] OR insufficienc*[Title/Abstract] OR descompensat*[Title/Abstract]) 279,638

#3 #1 or #2 310,240

#4 preserved ejection fraction[Title/Abstract] 6,959

#5 ("ejection fraction"[Title/Abstract] OR ef[Title/Abstract] OR lvef[Title/Abstract]) AND "preserved"[Title/Abstract] 11,377

#6 HFpEF[Title/Abstract] OR HF-pEF[Title/Abstract] 4,498

#7 #4 or #5 or #6 11,739

#8 "left ventricle"[Title/Abstract] AND "ejection fraction"[Title/Abstract] 6,850

#9 ("Ventricular Dysfunction, Left"[Mesh]) OR "Ventricular Function"[Mesh:NoExp] 46,718

#10 preserved[Title/Abstract] 121,989

#11 (#8 or #9) and #10 3,012

#12 (#7 or #11) and #3 9,187

#13 diagnosable[Title/Abstract] OR diagnosi[Title/Abstract] OR diagnosis[Title/Abstract] OR diagnose[Title/Abstract] OR diagnoses[Title/Abstract] OR diagnosed[Title/Abstract] OR diagnosing[Title/Abstract] OR theranostic[Title/Abstract] 2,453,174

#14 #12 and #13 1,633

#15 "decision making"[Title/Abstract] OR criteria*[Title/Abstract] OR tool*[Title/Abstract] OR algorithm*[Title/Abstract] 2,023,967

#16 #14 and #15 399

#17 #16 Filters: English, Spanish, from 2016/1/1 – 3000 280

**2. Initial bibliographical approach.**

**2.1. Introduction**

- Ho JE, Redfield MM, Lewis GD, Paulus WJ, Lam CSP. **Deliberating the Diagnostic Dilemma of Heart Failure With Preserved Ejection Fraction.** *Circulation*. 2020;142(18):1770–1780. doi:[10.1161/CIRCULATIONAHA.119.041818](https://doi.org/10.1161/CIRCULATIONAHA.119.041818). <https://www.ncbi.nlm.nih.gov/pubmed/33136513>
- Iyngkaran P, Thomas MC, Neil C, et al. **The Heart Failure with Preserved Ejection Fraction Conundrum-Redefining the Problem and Finding Common Ground?** *Curr Heart Fail Rep*. 2020;17(2):34–42. doi:[10.1007/s11897-020-00454-2](https://doi.org/10.1007/s11897-020-00454-2). <https://www.ncbi.nlm.nih.gov/pubmed/32112345>
- Thompson A., Crilley J., Wilson D., Hungin A.P.S., Fuat A., Murphy J. **An Epidemic of HFPEF?** *Heart*. 2016;102(Supplement 6):A15–A16. doi:[10.1136/heartjnl-2016-309890.24](https://doi.org/10.1136/heartjnl-2016-309890.24).
- Kapłon-Cieślicka A, Laroche C, Crespo-Leiro MG, et al. **Is heart failure misdiagnosed in hospitalized patients with preserved ejection fraction? From the European Society of Cardiology - Heart Failure Association  EURObservational Research Programme Heart Failure Long-Term Registry.** *ESC Heart Fail*. 2020;7(5):2098–2112. doi:[10.1002/ehf2.12817](https://doi.org/10.1002/ehf2.12817). <https://www.ncbi.nlm.nih.gov/pubmed/32618139>

**2.2. Diagnosis**

- Abramov D, Parwani P. **Diving Into the Diagnostic Score Algorithms of Heart Failure With Preserved Ejection Fraction.** *Front Cardiovasc Med*. 2021;8:665424. doi:[10.3389/fcvm.2021.665424](https://doi.org/10.3389/fcvm.2021.665424). <https://www.ncbi.nlm.nih.gov/pubmed/34179132>
- Adamczak DM, Oduah M-T, Kiebalo T, et al. **Heart Failure with Preserved Ejection Fraction-a Concise Review.** *Curr Cardiol Rep*. 2020;22(9):82. doi:[10.1007/s11886-020-01349-3](https://doi.org/10.1007/s11886-020-01349-3). <https://www.ncbi.nlm.nih.gov/pubmed/32648130>
- Al Saikhan L, Hughes AD, Chung W-S, Alsharqi M, Nihoyannopoulos P. **Left atrial function in heart failure with mid-range ejection fraction differs from that of heart failure with preserved ejection fraction: a 2D  speckle-tracking echocardiographic study.** *Eur Heart J Cardiovasc Imaging*. 2019;20(3):279–290. doi:[10.1093/ehjci/jey171](https://doi.org/10.1093/ehjci/jey171). <https://www.ncbi.nlm.nih.gov/pubmed/30517648>
- Amanai S, Harada T, Kagami K, et al. **The H(2)FPEF and HFA-PEFF algorithms for predicting exercise intolerance and abnormal hemodynamics in heart failure with preserved ejection fraction.** *Sci Rep*. 2022;12(1):13. doi:[10.1038/s41598-021-03974-6](https://doi.org/10.1038/s41598-021-03974-6). <https://www.ncbi.nlm.nih.gov/pubmed/34996984>
- Anderson T, Hummel SL, Konerman MC. **Epidemiology, Diagnosis, Pathophysiology, and Initial Approach to Heart Failure with Preserved Ejection Fraction.** *Cardiol Clin*. 2022;40(4):397–413. doi:[10.1016/j.ccl.2022.07.001](https://doi.org/10.1016/j.ccl.2022.07.001). <https://www.ncbi.nlm.nih.gov/pubmed/36210127>
- Araiza-Garaygordobil D, Fuentes-Mendoza A, Guerrero-Pando C, et al. **Heart failure with preserved ejection fraction: the dark side of an old disease.** *Arch Cardiol Mex*. 2019;89(4):360–368. doi:[10.24875/ACM.19000170](https://doi.org/10.24875/ACM.19000170). <https://www.ncbi.nlm.nih.gov/pubmed/31834308>
- Barandiarán Aizpurua A, Sanders-van Wijk S, Brunner-La Rocca H-P, et al. **Validation of the HFA-PEFF score for the diagnosis of heart failure with preserved ejection fraction.** *Eur J Heart Fail*. 2020;22(3):413–421. doi:[10.1002/ejhf.1614](https://doi.org/10.1002/ejhf.1614). <https://www.ncbi.nlm.nih.gov/pubmed/31472035>
- Baratto C., Caravita S., Sorropago A., et al. **Exercise echocardiography or cardiopulmonary exercise test to detect heart failure with preserved ejection fraction?** *J Hypertens*. 2019;37(Supplement 1):e113–e114.
- Baratto C, Caravita S, Soranna D, et al. **Current Limitations of Invasive Exercise Hemodynamics for the Diagnosis of Heart Failure With Preserved Ejection Fraction.** *Circ Heart Fail*. 2021;14(5):e007555. doi:[10.1161/CIRCHEARTFAILURE.120.007555](https://doi.org/10.1161/CIRCHEARTFAILURE.120.007555). <https://www.ncbi.nlm.nih.gov/pubmed/33951935>
- Barletta M., Bandera F., Palumbo M.C., et al. **Link between left ventricle diastole and right ventricle function: New insight into hypertensive patients developing heart failure with preserved ejection fraction**. *Eur Heart J Cardiovasc Imaging*. 2017;18(Supplement 3):iii402–iii403. doi:[10.1093/ehjci/jex299](https://doi.org/10.1093/ehjci/jex299).
- Buckley LF, Canada JM, Del Buono MG, et al. **Low NT-proBNP levels in overweight and obese patients do not rule out a diagnosis of heart failure with preserved ejection fraction.** *ESC Heart Fail*. 2018;5(2):372–378. doi:[10.1002/ehf2.12235](https://doi.org/10.1002/ehf2.12235). <https://www.ncbi.nlm.nih.gov/pubmed/29345112>
- Campbell DJ, Gong FF, Jelinek MV, et al. **Threshold body mass index and sex-specific waist circumference for increased risk of heart failure with preserved ejection fraction.** *Eur J Prev Cardiol*. 2019;26(15):1594–1602. doi:[10.1177/2047487319851298](https://doi.org/10.1177/2047487319851298). <https://www.ncbi.nlm.nih.gov/pubmed/31104485>
- Caravita S., Baratto C., Sorropago A., et al. **Exercise echocardiography or cardiopulmonary exercise test to detect pre-clinical heart failure with preserved ejection fraction?** *Eur Heart J*. 2018;39(Supplement 1):977–978. doi:[10.1093/eurheartj/ehy563.P4704](https://doi.org/10.1093/eurheartj/ehy563.P4704).
- Çavuşoğlu Y, Çelik A, Altay H, et al. **Heart failure with non-reduced ejection fraction: Epidemiology, pathophysiology, phenotypes, diagnosis and treatment approaches.** *Turk Kardiyol Dern Ars*. 2022;50(Supp1):S1–S34. doi:[10.5543/tkda.2022.S1](https://doi.org/10.5543/tkda.2022.S1). <https://www.ncbi.nlm.nih.gov/pubmed/35969235>
- Charman SJ, Okwose NC, Taylor CJ, et al. **Feasibility of the cardiac output response to stress test in suspected heart failure patients.** *Fam Pract*. 2022;39(5):805–812. doi:[10.1093/fampra/cmab184](https://doi.org/10.1093/fampra/cmab184). <https://www.ncbi.nlm.nih.gov/pubmed/35083480>
- Chiou Y-A, Hung C-L, Lin S-F. **AI-Assisted Echocardiographic Prescreening of Heart Failure With Preserved Ejection Fraction on the Basis of Intrabeat Dynamics.** *JACC Cardiovasc Imaging*. 2021;14(11):2091–2104. doi:[10.1016/j.jcmg.2021.05.005](https://doi.org/10.1016/j.jcmg.2021.05.005). <https://www.ncbi.nlm.nih.gov/pubmed/34147456>
- Cohen SS, Roger VL, Weston SA, et al. **Evaluation of claims-based computable phenotypes to identify heart failure patients with preserved ejection fraction.** *Pharmacol Res Perspect*. 2020;8(6):e00676. doi:[10.1002/prp2.676](https://doi.org/10.1002/prp2.676). <https://www.ncbi.nlm.nih.gov/pubmed/33124771>
- Dal Canto E, Remmelzwaal S, van Ballegooijen AJ, et al. **Diagnostic value of echocardiographic markers for diastolic dysfunction and heart failure with preserved ejection fraction.** *Heart Fail Rev*. 2022;27(1):207–218. doi:[10.1007/s10741-020-09985-1](https://doi.org/10.1007/s10741-020-09985-1). <https://www.ncbi.nlm.nih.gov/pubmed/32488580>
- Desai RJ, Lin KJ, Patorno E, et al. **Development and Preliminary Validation of a Medicare Claims-Based Model to Predict Left Ventricular Ejection Fraction Class in Patients With Heart Failure.** *Circ Cardiovasc Qual Outcomes*. 2018;11(12):e004700. doi:[10.1161/CIRCOUTCOMES.118.004700](https://doi.org/10.1161/CIRCOUTCOMES.118.004700). <https://www.ncbi.nlm.nih.gov/pubmed/30562067>
- Donal E. **The value of exercise echocardiography in heart failure with preserved ejection fraction.** *J Ultrason*. 2019;19(76):43–44. doi:[10.15557/JoU.2019.0005](https://doi.org/10.15557/JoU.2019.0005). <https://www.ncbi.nlm.nih.gov/pubmed/31088009>
- Escoli R, Carvalho MJ, Cabrita A, Rodrigues A. **Diastolic Dysfunction, an Underestimated New Challenge in Dialysis.** *Ther Apher Dial*. 2019;23(2):108–117. doi:[10.1111/1744-9987.12756](https://doi.org/10.1111/1744-9987.12756). <https://www.ncbi.nlm.nih.gov/pubmed/30255628>
- Ezekowitz JA, McAlister FA, Howlett J, et al. **A prospective evaluation of the established criteria for heart failure with preserved ejection fraction using the Alberta HEART cohort.** *ESC Heart Fail*. 2018;5(1):19–26. doi:[10.1002/ehf2.12200](https://doi.org/10.1002/ehf2.12200). <https://www.ncbi.nlm.nih.gov/pubmed/28741909>
- Faxen UL, Venkateshvaran A, Shah SJ, et al. **Generalizability of HFA-PEFF and H(2)FPEF Diagnostic Algorithms and Associations With Heart Failure Indices and Proteomic Biomarkers: Insights From PROMIS-HFpEF.** *J Card Fail*. 2021;27(7):756–765. doi:[10.1016/j.cardfail.2021.02.005](https://doi.org/10.1016/j.cardfail.2021.02.005). <https://www.ncbi.nlm.nih.gov/pubmed/33647474>
- Finet JE, Van Iterson EH, Wilson Tang WH. **Invasive Hemodynamic and Metabolic Evaluation of HFpEF.** *Curr Treat Options Cardiovasc Med*. 2021;23(5):32. doi:[10.1007/s11936-021-00904-7](https://doi.org/10.1007/s11936-021-00904-7). <https://www.ncbi.nlm.nih.gov/pubmed/34177247>
- Garg A, Virmani D, Agrawal S, et al. **Clinical Application of Biomarkers in Heart Failure with a Preserved Ejection Fraction: A Review.** *Cardiology*. 2017;136(3):192–203. doi:[10.1159/000450573](https://doi.org/10.1159/000450573). <https://www.ncbi.nlm.nih.gov/pubmed/27784010>
- Gevaert AB, Kataria R, Zannad F, et al. **Heart failure with preserved ejection fraction: recent concepts in diagnosis, mechanisms and management**. *Heart*. 2022;108(17):1342–1350. doi:[10.1136/heartjnl-2021-319605](https://doi.org/10.1136/heartjnl-2021-319605). <https://www.ncbi.nlm.nih.gov/pubmed/35022210>
- Gong A., Onwuzurike J.O., Balac N., Patel S.V., Sohn J., Grazette L.P. **SOLUBLE ST2 AND ITS ASSOCIATION WITH ECHOCARDIOGRAPHIC MEASURES OF DIASTOLIC DYSFUNCTION IN AMBULATORY PATIENTS WITH HFPEF**. *J Am Coll Cardiol*. 2021;77(18 Supplement 1):711. doi:[10.1016/S0735-1097%2821%2902070-2](https://doi.org/10.1016/S0735-1097%2821%2902070-2).
- Gregorova Z, Meluzin J, Stepanova R, Sitar J, Podrouzkova H, Spinarova L. **Longitudinal, circumferential and radial systolic left ventricular function in patients with heart failure and preserved ejection fraction.** *Biomed Pap Med Fac Univ Palacky Olomouc Czech Repub*. 2016;160(3):385–392. doi:[10.5507/bp.2016.007](https://doi.org/10.5507/bp.2016.007). <https://www.ncbi.nlm.nih.gov/pubmed/26948032>
- Guazzi M, Wilhelm M, Halle M, et al. **Exercise testing in heart failure with preserved ejection fraction: an appraisal through diagnosis, pathophysiology and therapy - A clinical consensus statement  of the Heart Failure Association and European Association of Preventive  Cardiology of the European Society of Cardiology.** *Eur J Heart Fail*. 2022;24(8):1327–1345. doi:[10.1002/ejhf.2601](https://doi.org/10.1002/ejhf.2601). <https://www.ncbi.nlm.nih.gov/pubmed/35775383>
- Gudieva K., Islamova M., Soloveva A., et al. **Incidence and probability of heart failure with preserved ejection fraction among high risk hypertensive patients**. *Eur J Heart Fail*. 2019;21(Supplement 1):389–390. doi:[10.1002/ejhf.1488](https://doi.org/10.1002/ejhf.1488).
- Hafez MS, El Missiri AM. **Left Atrial Ejection Force as a Marker for the Diagnosis of Heart Failure with Preserved Ejection Fraction.** *J Cardiovasc Echogr*. 2021;31(3):125–130. doi:[10.4103/jcecho.jcecho_142_20](https://doi.org/10.4103/jcecho.jcecho_142_20). <https://www.ncbi.nlm.nih.gov/pubmed/34900546>
- Hage C, LÖfstrÖm U, Donal E, et al. **Do Patients With Acute Heart Failure and Preserved Ejection Fraction Have Heart Failure at Follow-Up: Implications of the Framingham Criteria.** *J Card Fail*. 2020;26(8):673–684. doi:[10.1016/j.cardfail.2019.04.013](https://doi.org/10.1016/j.cardfail.2019.04.013). <https://www.ncbi.nlm.nih.gov/pubmed/31035008>
- Hagendorff A, Helfen A, Brandt R, et al. **Expert proposal to characterize cardiac diseases with normal or preserved left ventricular ejection fraction and symptoms of heart failure by comprehensive  echocardiography.** *Clin Res Cardiol*. 2022. doi:[10.1007/s00392-022-02041-y](https://doi.org/10.1007/s00392-022-02041-y). <https://www.ncbi.nlm.nih.gov/pubmed/35660948>
- Hagendorff A, Stöbe S, Kandels J, de Boer R, Tschöpe C. **Diagnostic role of echocardiography for patients with heart failure symptoms and preserved left ventricular ejection fraction.** *Herz*. 2022;47(4):293–300. doi:[10.1007/s00059-022-05118-6](https://doi.org/10.1007/s00059-022-05118-6). <https://www.ncbi.nlm.nih.gov/pubmed/35499562>
- Hassan OKA, Higgins AR. **The role of multimodality imaging in patients with heart failure with reduced and preserved ejection fraction.** *Curr Opin Cardiol*. 2022;37(3):285–293. doi:[10.1097/HCO.0000000000000963](https://doi.org/10.1097/HCO.0000000000000963). <https://www.ncbi.nlm.nih.gov/pubmed/35612940>
- He J, Yang W, Jiang Y, et al. **Heart failure with preserved ejection fraction assessed by cardiac magnetic resonance: From clinical uses to emerging techniques.** *Trends Cardiovasc Med*. 2021:S1050-1738(21)00156–0. doi:[10.1016/j.tcm.2021.12.006](https://doi.org/10.1016/j.tcm.2021.12.006). <https://www.ncbi.nlm.nih.gov/pubmed/34933114>
- Henkens MTHM, van Ommen A-M, Remmelzwaal S, et al. **The HFA-PEFF score identifies “early-HFpEF” phenogroups associated with distinct biomarker profiles.** *ESC Heart Fail*. 2022;9(3):2032–2036. doi:[10.1002/ehf2.13861](https://doi.org/10.1002/ehf2.13861). <https://www.ncbi.nlm.nih.gov/pubmed/35301820>
- Henning RJ. **Diagnosis and treatment of heart failure with preserved left ventricular ejection fraction.** *World J Cardiol*. 2020;12(1):7–25. doi:[10.4330/wjc.v12.i1.7](https://doi.org/10.4330/wjc.v12.i1.7). <https://www.ncbi.nlm.nih.gov/pubmed/31984124>
- Huis In ’t Veld AE, de Man FS, van Rossum AC, Handoko ML. **How to diagnose heart failure with preserved ejection fraction: the value of invasive stress testing.** *Neth Heart J*. 2016;24(4):244–251. doi:[10.1007/s12471-016-0811-0](https://doi.org/10.1007/s12471-016-0811-0). <https://www.ncbi.nlm.nih.gov/pubmed/26914917>
- Huusko J, Purmonen T, Toppila I, Lassenius M, Ukkonen H. **Real-world clinical diagnostics of heart failure patients with reduced or preserved ejection fraction.** *ESC Heart Fail*. 2020;7(3):1039–1048. doi:[10.1002/ehf2.12665](https://doi.org/10.1002/ehf2.12665). <https://www.ncbi.nlm.nih.gov/pubmed/32187879>
- Hwang I-C, Cho G-Y, Choi H-M, et al. **H2FPEF Score Reflects the Left Atrial Strain and Predicts Prognosis in Patients With Heart Failure With Preserved Ejection Fraction.** *J Card Fail*. 2021;27(2):198–207. doi:[10.1016/j.cardfail.2020.09.474](https://doi.org/10.1016/j.cardfail.2020.09.474). <https://www.ncbi.nlm.nih.gov/pubmed/33035685>
- Johansson MC, Rosengren A, Fu M. **Echocardiographic diagnosis of heart failure with preserved ejection fraction in elderly patients with hypertension.** *Scand Cardiovasc J*. 2022;56(1):368–377. doi:[10.1080/14017431.2022.2129777](https://doi.org/10.1080/14017431.2022.2129777). <https://www.ncbi.nlm.nih.gov/pubmed/36266943>
- Kagami K, Harada T, Yoshida K, et al. **Impaired Right Atrial Reserve Function in Heart Failure with Preserved Ejection Fraction.** *J Am Soc Echocardiogr*. 2022;35(8):836–845. doi:[10.1016/j.echo.2022.03.006](https://doi.org/10.1016/j.echo.2022.03.006). <https://www.ncbi.nlm.nih.gov/pubmed/35283241>
- Kapłon-Cieślicka A, Lund LH. **Do we need a definition of acute heart failure with preserved ejection fraction?** *Ann Med*. 2021;53(1):1470–1475. doi:[10.1080/07853890.2021.1968028](https://doi.org/10.1080/07853890.2021.1968028). <https://www.ncbi.nlm.nih.gov/pubmed/34431429>
- Kosmala W, Przewlocka-Kosmala M, Rojek A, Marwick TH. **Comparison of the Diastolic Stress Test With a Combined Resting Echocardiography and Biomarker Approach to Patients With Exertional Dyspnea: Diagnostic and  Prognostic Implications.** *JACC Cardiovasc Imaging*. 2019;12(5):771–780. doi:[10.1016/j.jcmg.2017.10.008](https://doi.org/10.1016/j.jcmg.2017.10.008). <https://www.ncbi.nlm.nih.gov/pubmed/29454783>
- Larina V.N., Lunev V.I. **The value of biomarkers in the diagnosis and prognosis of heart failure in older age**. *Russian Arch Int Med*. 2021;11(2):98–110. doi:[10.20514/2226-6704-2021-11-2-98-110](https://doi.org/10.20514/2226-6704-2021-11-2-98-110).
- Lin H, Hartley P, Forsyth F, et al. **Clinical and demographic correlates of accelerometer-measured physical activity in participants enrolled in the OPTIMISE HFpEF study.** *Eur J Cardiovasc Nurs*. 2022;21(1):67–75. doi:[10.1093/eurjcn/zvab028](https://doi.org/10.1093/eurjcn/zvab028). <https://www.ncbi.nlm.nih.gov/pubmed/33837414>
- Lin T-T, Wang Y-C, Juang J-MJ, Hwang J-J, Wu C-K. **Application of the newest European Association of Cardiovascular Imaging Recommendation regarding the long-term prognostic relevance of left ventricular  diastolic function in heart failure with preserved ejection fraction.** *Eur Radiol*. 2020;30(1):630–639. doi:[10.1007/s00330-019-06261-1](https://doi.org/10.1007/s00330-019-06261-1). <https://www.ncbi.nlm.nih.gov/pubmed/31396729>
- Liu S, Iskandar R, Chen W, et al. **Soluble Glycoprotein 130 and Heat Shock Protein 27 as Novel Candidate Biomarkers of Chronic Heart Failure with Preserved Ejection Fraction.** *Heart Lung Circ*. 2016;25(10):1000–1006. doi:[10.1016/j.hlc.2016.02.011](https://doi.org/10.1016/j.hlc.2016.02.011). <https://www.ncbi.nlm.nih.gov/pubmed/27067668>
- Liu Y, Guo X, Zheng Y. **An Automatic Approach Using ELM Classifier for HFpEF Identification Based on Heart Sound Characteristics.** *J Med Syst*. 2019;43(9):285. doi:[10.1007/s10916-019-1415-1](https://doi.org/10.1007/s10916-019-1415-1). <https://www.ncbi.nlm.nih.gov/pubmed/31309299>
- Loai S, Cheng H-LM. **Heart failure with preserved ejection fraction: the missing pieces in diagnostic imaging.** *Heart Fail Rev*. 2020;25(2):305–319. doi:[10.1007/s10741-019-09836-8](https://doi.org/10.1007/s10741-019-09836-8). <https://www.ncbi.nlm.nih.gov/pubmed/31364028>
- Logeart D., Paven E., Damy T., et al. **Imaging criteria for the diagnosis of heart failure with midrange and preserved LVEF in the real life**. *Eur Heart J*. 2019;40(Supplement 1):50. doi:[10.1093/eurheartj/ehz747.0048](https://doi.org/10.1093/eurheartj/ehz747.0048).
- Lotierzo M, Bruno R, Finan-Marchi A, et al. **Could a Multi-Marker and Machine Learning Approach Help Stratify Patients with Heart Failure?** *Medicina (Kaunas)*. 2021;57(10). doi:[10.3390/medicina57100996](https://doi.org/10.3390/medicina57100996). <https://www.ncbi.nlm.nih.gov/pubmed/34684033>
- Ma C-S, Liao Y-P, Fan J-L, Zhao X, Su B, Zhou B-Y. **The novel left atrial strain parameters in diagnosing of heart failure with preserved ejection fraction.** *Echocardiography*. 2022;39(3):416–425. doi:[10.1111/echo.15304](https://doi.org/10.1111/echo.15304). <https://www.ncbi.nlm.nih.gov/pubmed/35076951>
- Ma C, Luo H, Fan L, Liu X, Gao C. **Heart failure with preserved ejection fraction: an update on pathophysiology, diagnosis, treatment, and prognosis.** *Braz J Med Biol Res*. 2020;53(7):e9646. doi:[10.1590/1414-431X20209646](https://doi.org/10.1590/1414-431X20209646). <https://www.ncbi.nlm.nih.gov/pubmed/32520204>
- Mandoli GE, Sisti N, Mondillo S, Cameli M. **Left atrial strain in left ventricular diastolic dysfunction: have we finally found the missing piece of the puzzle?** *Heart Fail Rev*. 2020;25(3):409–417. doi:[10.1007/s10741-019-09889-9](https://doi.org/10.1007/s10741-019-09889-9). <https://www.ncbi.nlm.nih.gov/pubmed/31773504>
- Manukyan MA, Falkovskaya AY, Zyubanova IV, et al. **Diastolic Dysfunction and Heart Failure With Preserved Ejection Fraction In Patients With Resistant Hypertension and Type 2 Diabetes Mellitus.** *Kardiologiia*. 2022;62(8):11–18. doi:[10.18087/cardio.2022.8.n1706](https://doi.org/10.18087/cardio.2022.8.n1706). <https://www.ncbi.nlm.nih.gov/pubmed/36066982>
- Marta Szabo M., Muk B., Majoros Z.S., et al. **The importance of the NTproBNP rule-in criteria in the early diagnosis of HFmrEF and HFpEF**. *Eur J Heart Fail*. 2018;20(Supplement 1):433. doi:[10.1002/ejhf.1197](https://doi.org/10.1002/ejhf.1197).
- Martinez-Marin M., Josa-Laorden C., Lacambra-Blasco I., et al. **Diagnostic and prognostic role of the myocardial deformation analysis in heart failure**. *Eur Heart J*. 2016;37(Supplement 1):254. doi:[10.1093/eurheartj/ehw432](https://doi.org/10.1093/eurheartj/ehw432).
- Miguel Martinez-Marin M., Josa-Laorden C., Rubio-Gracia J., et al. **Utility of myocardial deformation analysis in the heart failure**. *Eur J Heart Fail*. 2016;18(SUPPL. 1):390. doi:[10.1002/ejhf.539](https://doi.org/10.1002/ejhf.539).
- Mitter SS, Shah SJ, Thomas JD. **A Test in Context: E/A and E/e’ to Assess Diastolic Dysfunction and LV Filling Pressure.** *J Am Coll Cardiol*. 2017;69(11):1451–1464. doi:[10.1016/j.jacc.2016.12.037](https://doi.org/10.1016/j.jacc.2016.12.037). <https://www.ncbi.nlm.nih.gov/pubmed/28302294>
- Mizobuchi A, Osawa K, Tanaka M, Yumoto A, Saito H, Fuke S. **Detrended fluctuation analysis can detect the impairment of heart rate regulation in patients with heart failure with preserved ejection fraction.** *J Cardiol*. 2021;77(1):72–78. doi:[10.1016/j.jjcc.2020.07.027](https://doi.org/10.1016/j.jjcc.2020.07.027). <https://www.ncbi.nlm.nih.gov/pubmed/32826140>
- Mordi IR, Singh S, Rudd A, et al. **Comprehensive Echocardiographic and Cardiac Magnetic Resonance Evaluation Differentiates Among Heart Failure With Preserved Ejection Fraction Patients,  Hypertensive Patients, and Healthy Control Subjects.** *JACC Cardiovasc Imaging*. 2018;11(4):577–585. doi:[10.1016/j.jcmg.2017.05.022](https://doi.org/10.1016/j.jcmg.2017.05.022). <https://www.ncbi.nlm.nih.gov/pubmed/28823736>
- Morfino P, Aimo A, Castiglione V, Vergaro G, Emdin M, Clerico A. **Biomarkers of HFpEF: Natriuretic Peptides, High-Sensitivity Troponins and Beyond.** *J Cardiovasc Dev Dis*. 2022;9(8). doi:[10.3390/jcdd9080256](https://doi.org/10.3390/jcdd9080256). <https://www.ncbi.nlm.nih.gov/pubmed/36005420>
- Morvai-Illés B, Polestyuk-Németh N, Szabó IA, et al. **The Prognostic Value of Lung Ultrasound in Patients With Newly Diagnosed Heart Failure With Preserved Ejection Fraction in the Ambulatory Setting.** *Front Cardiovasc Med*. 2021;8:758147. doi:[10.3389/fcvm.2021.758147](https://doi.org/10.3389/fcvm.2021.758147). <https://www.ncbi.nlm.nih.gov/pubmed/34926610>
- Nagueh SF. **Diagnostic Algorithms for Heart Failure With Preserved Ejection Fraction.** *JACC Heart Fail*. 2020;8(8):654–656. doi:[10.1016/j.jchf.2020.04.005](https://doi.org/10.1016/j.jchf.2020.04.005). <https://www.ncbi.nlm.nih.gov/pubmed/32535120>
- Nagueh SF. ***Heart failure with preserved ejection fraction: insights into diagnosis and pathophysiology****.* England; 2021. doi:[10.1093/cvr/cvaa228](https://doi.org/10.1093/cvr/cvaa228).
- Neves B., Hammoudi N., Pousset F., et al. **Echocardiographic diagnostic criteria for heart failure with preserved ejection fraction: Does one-size fits all?** *Eur J Heart Fail*. 2017;19(Supplement 1):221–222. doi:[10.1002/ejhf.833](https://doi.org/10.1002/ejhf.833).
- Nguyen T., Duong K., Tuyet Nhi L.N., Tri Nhan N.M., Phuoc L.T., Rigatelli G. **New and definitive marker confirming the optimal status in the treatment of heart failure with preserved ejection fraction**. *J Am Coll Cardiol*. 2017;70(16 Supplement 1):C147. doi:[10.1016/j.jacc.2017.07.530](https://doi.org/10.1016/j.jacc.2017.07.530).
- Nielsen OW, Valeur N, Sajadieh A, Fabricius-Bjerre A, Carlsen CM, Kober L. **Echocardiographic subtypes of heart failure in consecutive hospitalised patients with dyspnoea.** *Open Heart*. 2019;6(1):e000928. doi:[10.1136/openhrt-2018-000928](https://doi.org/10.1136/openhrt-2018-000928). <https://www.ncbi.nlm.nih.gov/pubmed/31297224>
- Nikorowitsch J., Bei Der Kellen R., Kirchhof P., et al. **Applying the ESC 2016, the H2FPEF, and the HFA-PEFF diagnostic algorithms for heart failure with preserved ejection fraction to the general population - a comparative approach**. *Eur Heart J*. 2021;42(SUPPL 1):856. doi:[10.1093/eurheartj/ehab724.0856](https://doi.org/10.1093/eurheartj/ehab724.0856).
- Nikorowitsch J, Bei der Kellen R, Kirchhof P, et al. **Applying the ESC 2016, H(2) FPEF, and HFA-PEFF diagnostic algorithms for heart failure with preserved ejection fraction to the general population.** *ESC Heart Fail*. 2021;8(5):3603–3612. doi:[10.1002/ehf2.13532](https://doi.org/10.1002/ehf2.13532). <https://www.ncbi.nlm.nih.gov/pubmed/34459154>
- Oakland HT, Joseph P, Elassal A, Cullinan M, Heerdt PM, Singh I. **Diagnostic utility of sub-maximum cardiopulmonary exercise testing in the ambulatory setting for heart failure with preserved ejection fraction.** *Pulm Circ*. 2020;10(4):2045894020972273. doi:[10.1177/2045894020972273](https://doi.org/10.1177/2045894020972273). <https://www.ncbi.nlm.nih.gov/pubmed/33282205>
- Okada M., Inoue K., Iwakura K., et al. **Mechanism and Prognostic Significance of Cardiothoracic Ratio in Patients with Heart Failure with Preserved Ejection Fraction - Insights from Pursuit-hfpef Registry - Insights f**. *Circulation*. 2020;142(SUPPL 3). doi:[10.1161/circ.142.suppl_3.13493](https://doi.org/10.1161/circ.142.suppl_3.13493).
- Okada M., Tanaka N., Toshinari O., et al. **Left Atrial Volumetric/Mechanical Coupling Index: A Novel Predictor of Clinical Outcomes in Heart Failure With Preserved Ejection Fraction**. *Circulation*. 2022;146(Supplement 1). doi:[10.1161/circ.146.suppl_1.11223](https://doi.org/10.1161/circ.146.suppl_1.11223).
- Ozen Kavas P., Recep Bozkurt M., Kocayigit I., Bilgin C. **Machine learning-based medical decision support system for diagnosing HFpEF and HFrEF using PPG**. *Biomed Signal Process Control*. 2023;79((Ozen Kavas) Computer Engineering, Faculty of Engineering, Kutahya Dumlupinar University, Kutahya, Turkey):104164. doi:[10.1016/j.bspc.2022.104164](https://doi.org/10.1016/j.bspc.2022.104164).
- Pabón MA, Cunningham JW, Claggett BL, et al. **Natriuretic peptide-based inclusion criteria in heart failure with preserved ejection fraction clinical trials: insights from PARAGON-HF.** *Eur J Heart Fail*. 2022;24(4):672–677. doi:[10.1002/ejhf.2439](https://doi.org/10.1002/ejhf.2439). <https://www.ncbi.nlm.nih.gov/pubmed/35080787>
- Palazzuoli A, Caravita S, Paolillo S, et al. **Current gaps in HFpEF trials: Time to reconsider patients’ selection and to target phenotypes.** *Prog Cardiovasc Dis*. 2021;67:89–97. doi:[10.1016/j.pcad.2021.03.007](https://doi.org/10.1016/j.pcad.2021.03.007). <https://www.ncbi.nlm.nih.gov/pubmed/33839166>
- Parcha V, Malla G, Kalra R, et al. **Diagnostic and prognostic implications of heart failure with preserved ejection fraction scoring systems.** *ESC Heart Fail*. 2021;8(3):2089–2102. doi:[10.1002/ehf2.13288](https://doi.org/10.1002/ehf2.13288). <https://www.ncbi.nlm.nih.gov/pubmed/33709628>
- Parvan R, Hosseinpour M, Moradi Y, Devaux Y, Cataliotti A, da Silva GJJ. **Diagnostic performance of microRNAs in the detection of heart failure with reduced or preserved ejection fraction: a systematic review and meta-analysis.** *Eur J Heart Fail*. 2022. doi:[10.1002/ejhf.2700](https://doi.org/10.1002/ejhf.2700). <https://www.ncbi.nlm.nih.gov/pubmed/36161443>
- Patel R, Desai AR, Patel P, Vanka V, Ghadiam H, Kizhakekuttu T. **Physician Variability in Diastology Reporting in Patients With Preserved Ejection Fraction: A Single Center Experience.** *Cureus*. 2020;12(7):e9062. doi:[10.7759/cureus.9062](https://doi.org/10.7759/cureus.9062). <https://www.ncbi.nlm.nih.gov/pubmed/32782880>
- Patel RB, Shah SJ. **Inclusion Criteria for Heart Failure With Preserved Ejection Fraction Clinical Trials: Making the Case for Precision Diagnosis and Greater Inclusivity.** *J Card Fail*. 2022;28(5):732–735. doi:[10.1016/j.cardfail.2022.03.345](https://doi.org/10.1016/j.cardfail.2022.03.345). <https://www.ncbi.nlm.nih.gov/pubmed/35331890>
- Patel YR, Robbins JM, Kurgansky KE, et al. **Development and validation of a heart failure with preserved ejection fraction cohort using electronic medical records.** *BMC Cardiovasc Disord*. 2018;18(1):128. doi:[10.1186/s12872-018-0866-5](https://doi.org/10.1186/s12872-018-0866-5). <https://www.ncbi.nlm.nih.gov/pubmed/29954337>
- Persson H, Donal E, Lund LH, et al. **Importance of structural heart disease and diastolic dysfunction in heart failure with preserved ejection fraction assessed according to the ESC guidelines - A  substudy in the Ka (Karolinska) Ren (Rennes) study.** *Int J Cardiol*. 2019;274:202–207. doi:[10.1016/j.ijcard.2018.06.078](https://doi.org/10.1016/j.ijcard.2018.06.078). <https://www.ncbi.nlm.nih.gov/pubmed/30049496>
- Pieske B, Tschöpe C, de Boer RA, et al. **How to diagnose heart failure with preserved ejection fraction: the HFA-PEFF diagnostic algorithm: a consensus recommendation from the Heart Failure  Association (HFA) of the European Society of Cardiology (ESC).** *Eur Heart J*. 2019;40(40):3297–3317. doi:[10.1093/eurheartj/ehz641](https://doi.org/10.1093/eurheartj/ehz641). <https://www.ncbi.nlm.nih.gov/pubmed/31504452>
- Pieske B, Tschöpe C, de Boer RA, et al. **How to diagnose heart failure with preserved ejection fraction: the HFA-PEFF diagnostic algorithm: a consensus recommendation from the Heart Failure  Association (HFA) of the European Society of Cardiology (ESC).** *Eur J Heart Fail*. 2020;22(3):391–412. doi:[10.1002/ejhf.1741](https://doi.org/10.1002/ejhf.1741). <https://www.ncbi.nlm.nih.gov/pubmed/32133741>
- Pocock SJ, Ferreira JP, Packer M, et al. **Biomarker-driven prognostic models in chronic heart failure with preserved ejection fraction: the EMPEROR-Preserved trial.** *Eur J Heart Fail*. 2022;24(10):1869–1878. doi:[10.1002/ejhf.2607](https://doi.org/10.1002/ejhf.2607). <https://www.ncbi.nlm.nih.gov/pubmed/35796209>
- Prasad SB, Holland DJ, Atherton JJ, Whalley G. **New Diastology Guidelines: Evolution, Validation and Impact on Clinical Practice.** *Heart Lung Circ*. 2019;28(9):1411–1420. doi:[10.1016/j.hlc.2019.03.013](https://doi.org/10.1016/j.hlc.2019.03.013). <https://www.ncbi.nlm.nih.gov/pubmed/31064714>
- Przewlocka-Kosmala M, Butler J, Donal E, Ponikowski P, Kosmala W. **Prognostic Value of the MAGGIC Score, H(2)FPEF Score, and HFA-PEFF Algorithm in Patients with Exertional Dyspnea and the Incremental Value of Exercise  Echocardiography.** *J Am Soc Echocardiogr*. 2022;35(9):966–975. doi:[10.1016/j.echo.2022.05.006](https://doi.org/10.1016/j.echo.2022.05.006). <https://www.ncbi.nlm.nih.gov/pubmed/35605894>
- Reddy YNV, Carter RE, Obokata M, Redfield MM, Borlaug BA. **A Simple, Evidence-Based Approach to Help Guide Diagnosis of Heart Failure With Preserved Ejection Fraction.** *Circulation*. 2018;138(9):861–870. doi:[10.1161/CIRCULATIONAHA.118.034646](https://doi.org/10.1161/CIRCULATIONAHA.118.034646). <https://www.ncbi.nlm.nih.gov/pubmed/29792299>
- Rendón-Giraldo JA, Lema C, Saldarriaga-Giraldo CI. **[Association between diastolic stress test and H2FPEF score].** *Arch Cardiol Mex*. 2022;92(2):203–208. doi:[10.24875/ACM.21000053](https://doi.org/10.24875/ACM.21000053). <https://www.ncbi.nlm.nih.gov/pubmed/34320623>
- Rimbas RC, Visoiu IS, Magda SL, et al. **New insights into the potential utility of the left atrial function analysis in heart failure with preserved ejection fraction diagnosis.** *PLoS One*. 2022;17(5):e0267962. doi:[10.1371/journal.pone.0267962](https://doi.org/10.1371/journal.pone.0267962). <https://www.ncbi.nlm.nih.gov/pubmed/35507565>
- Rosch S, Kresoja K-P, Besler C, et al. **Characteristics of Heart Failure With Preserved Ejection Fraction Across the Range of Left Ventricular Ejection Fraction**. *Circulation*. 2022;146(7):506–518. doi:[10.1161/CIRCULATIONAHA.122.059280](https://doi.org/10.1161/CIRCULATIONAHA.122.059280). <https://www.ncbi.nlm.nih.gov/pubmed/35862208>
- Sanchez-Martinez S, Duchateau N, Erdei T, et al. **Machine Learning Analysis of Left Ventricular Function to Characterize Heart Failure With Preserved Ejection Fraction.** *Circ Cardiovasc Imaging*. 2018;11(4):e007138. doi:[10.1161/CIRCIMAGING.117.007138](https://doi.org/10.1161/CIRCIMAGING.117.007138). <https://www.ncbi.nlm.nih.gov/pubmed/29661795>
- Sanna GD, Canonico ME, Santoro C, et al. **Echocardiographic Longitudinal Strain Analysis in Heart Failure: Real Usefulness for Clinical Management Beyond Diagnostic Value and Prognostic Correlations? A  Comprehensive Review.** *Curr Heart Fail Rep*. 2021;18(5):290–303. doi:[10.1007/s11897-021-00530-1](https://doi.org/10.1007/s11897-021-00530-1). <https://www.ncbi.nlm.nih.gov/pubmed/34398411>
- Saxon DT, Kennel PJ, Guyer HM, Goyal P, Hummel SL, Konerman MC. **Specialty-Based Variability in Diagnosing and Managing Heart Failure With Preserved Ejection Fraction.** *Mayo Clin Proc*. 2020;95(4):669–675. doi:[10.1016/j.mayocp.2019.09.026](https://doi.org/10.1016/j.mayocp.2019.09.026). <https://www.ncbi.nlm.nih.gov/pubmed/32247341>
- Sbarbaro J.A., Hardin K., Farrell R., et al. **Upright exercise hemodynamic profiles effectively risk stratify patients with heart failure with preserved ejection fraction and elevated resting filling pressures**. *Circulation*. 2019;140(Supplement 1). doi:[10.1161/circ.140.suppl_1.15124](https://doi.org/10.1161/circ.140.suppl_1.15124).
- Schott A, Kluttig A, Mikolajczyk R, et al. **Association of arterial stiffness and heart failure with preserved ejection fraction in the elderly population - results from the CARLA study.** *J Hum Hypertens*. 2022. doi:[10.1038/s41371-022-00703-y](https://doi.org/10.1038/s41371-022-00703-y). <https://www.ncbi.nlm.nih.gov/pubmed/35581324>
- Selvaraj S, Myhre PL, Vaduganathan M, et al. **Application of Diagnostic Algorithms for Heart Failure With Preserved Ejection Fraction to the Community.** *JACC Heart Fail*. 2020;8(8):640–653. doi:[10.1016/j.jchf.2020.03.013](https://doi.org/10.1016/j.jchf.2020.03.013). <https://www.ncbi.nlm.nih.gov/pubmed/32535127>
- Seo Y. **Diagnosis of heart failure in the elderly: current status and future perspectives for echocardiographic diagnostic systems.** *J Med Ultrason (2001)*. 2022;49(3):381–388. doi:[10.1007/s10396-022-01223-5](https://doi.org/10.1007/s10396-022-01223-5). <https://www.ncbi.nlm.nih.gov/pubmed/35759116>
- Seo Y, Ishizu T, Ieda M, Ohte N. **Clinical Usefulness of the HFA-PEFF Diagnostic Scoring System in Identifying Late Elderly Heart Failure With Preserved Ejection Fraction Patients.** *Circ J*. 2021;85(5):604–611. doi:[10.1253/circj.CJ-20-0784](https://doi.org/10.1253/circj.CJ-20-0784). <https://www.ncbi.nlm.nih.gov/pubmed/33250499>
- Shah SJ. **BNP: Biomarker Not Perfect in heart failure with preserved ejection fraction**. *Eur Heart J*. 2022;43(20):1952–1954. doi:[10.1093/eurheartj/ehac121](https://doi.org/10.1093/eurheartj/ehac121). https://www.ncbi.nlm.nih.gov/pubmed/35301541
- Silbiger JJ. **Pathophysiology and Echocardiographic Diagnosis of Left Ventricular Diastolic Dysfunction.** *J Am Soc Echocardiogr*. 2019;32(2):216-232.e2. doi:[10.1016/j.echo.2018.11.011](https://doi.org/10.1016/j.echo.2018.11.011). <https://www.ncbi.nlm.nih.gov/pubmed/30717860>
- Spinarova M. **Noninvasive assessment of heart failure with preserved ejection fraction: New results in echocardiographic measurment of diastolic stiffness and intraventricular vortex**. *Eur Heart J*. 2016;37(Supplement 1):719. doi:[10.1093/eurheartj/ehw433](https://doi.org/10.1093/eurheartj/ehw433).
- Spinarova M., Meluzin J., Podrouzkova H., Stepanova R., Spinarova L. **Diastolic wall strain and vortex formation time: New parameters in assessment of heart failure with preserved ejection fraction**. *Eur J Heart Fail*. 2016;18(SUPPL. 1):198. doi:[10.1002/ejhf.539](https://doi.org/10.1002/ejhf.539).
- Špinarová M, Meluzín J, Podroužková H, Štěpánová R, Špinarová L. **New echocardiographic parameters in the diagnosis of heart failure with preserved ejection fraction.** *Int J Cardiovasc Imaging*. 2018;34(2):229–235. doi:[10.1007/s10554-017-1230-z](https://doi.org/10.1007/s10554-017-1230-z). <https://www.ncbi.nlm.nih.gov/pubmed/28819869>
- Spyridon-Michail Papazoglou S.-M., Jaffer U., Aslam M., et al. **Do NT-proBNP levels depend on the diameter of abdominal aorta in patients with chronic HFpEF and AAA?** *Eur J Heart Fail*. 2019;21(Supplement 1):51–52. doi:[10.1002/ejhf.1488](https://doi.org/10.1002/ejhf.1488).
- Stringer B., Hyder S., Nozadze N., Harmon R., Henry L., Chen K. **Analysis Of Clinical, Biochemical And Echocardiographic Criteria For Heart Failure With Preserved Ejection Fraction**. *J Card Fail*. 2022;28(5 Supplement):S107. doi:[10.1016/j.cardfail.2022.03.273](https://doi.org/10.1016/j.cardfail.2022.03.273).
- Sugimoto T. **Acute Decompensated Heart Failure in Patients with Heart Failure with Preserved Ejection Fraction.** *Heart Fail Clin*. 2020;16(2):201–209. doi:[10.1016/j.hfc.2019.12.002](https://doi.org/10.1016/j.hfc.2019.12.002). <https://www.ncbi.nlm.nih.gov/pubmed/32143764>
- Sun Y, Si J, Li J, et al. **Predictive Value of HFA-PEFF Score in Patients With Heart Failure With Preserved Ejection Fraction.** *Front Cardiovasc Med*. 2021;8:656536. doi:[10.3389/fcvm.2021.656536](https://doi.org/10.3389/fcvm.2021.656536). <https://www.ncbi.nlm.nih.gov/pubmed/34778384>
- Sun Y, Wang N, Li X, et al. **Predictive value of H(2) FPEF score in patients with heart failure with preserved ejection fraction.** *ESC Heart Fail*. 2021;8(2):1244–1252. doi:[10.1002/ehf2.13187](https://doi.org/10.1002/ehf2.13187). <https://www.ncbi.nlm.nih.gov/pubmed/33403825>
- Sundaram V, Zakeri R, Witte KK, Quint JK. **Development of algorithms for determining heart failure with reduced and preserved ejection fraction using nationwide electronic healthcare records in the  UK.** *Open Heart*. 2022;9(2). doi:[10.1136/openhrt-2022-002142](https://doi.org/10.1136/openhrt-2022-002142). <https://www.ncbi.nlm.nih.gov/pubmed/36332942>
- Sunderji I, Singh V, Fraser AG. **When does the E/e’ index not work? The pitfalls of oversimplifying diastolic function.** *Echocardiography*. 2020;37(11):1897–1907. doi:[10.1111/echo.14697](https://doi.org/10.1111/echo.14697). <https://www.ncbi.nlm.nih.gov/pubmed/32608167>
- Tabassian M, Sunderji I, Erdei T, et al. **Diagnosis of Heart Failure With Preserved Ejection Fraction: Machine Learning of Spatiotemporal Variations in Left Ventricular Deformation.** *J Am Soc Echocardiogr*. 2018;31(12):1272-1284.e9. doi:[10.1016/j.echo.2018.07.013](https://doi.org/10.1016/j.echo.2018.07.013). <https://www.ncbi.nlm.nih.gov/pubmed/30146187>
- Tada A, Nagai T, Omote K, et al. **Performance of the H(2)FPEF and the HFA-PEFF scores for the diagnosis of heart failure with preserved ejection fraction in Japanese patients: A report from the  Japanese multicenter registry.** *Int J Cardiol*. 2021;342:43–48. doi:[10.1016/j.ijcard.2021.08.001](https://doi.org/10.1016/j.ijcard.2021.08.001). <https://www.ncbi.nlm.nih.gov/pubmed/34364907>
- Tadic M, Cuspidi C, Calicchio F, Grassi G, Mancia G. **Diagnostic algorithm for HFpEF: how much is the recent consensus applicable in clinical practice?** *Heart Fail Rev*. 2021;26(6):1485–1493. doi:[10.1007/s10741-020-09966-4](https://doi.org/10.1007/s10741-020-09966-4). <https://www.ncbi.nlm.nih.gov/pubmed/32346825>
- Tadic M, Pieske-Kraigher E, Cuspidi C, et al. **Left ventricular strain and twisting in heart failure with preserved ejection fraction: an updated review.** *Heart Fail Rev*. 2017;22(3):371–379. doi:[10.1007/s10741-017-9618-3](https://doi.org/10.1007/s10741-017-9618-3). <https://www.ncbi.nlm.nih.gov/pubmed/28405789>
- Tan TS, Akbulut IM, Demirtola AI, et al. **LA reservoir strain: a sensitive parameter for estimating LV filling pressure in patients with preserved EF.** *Int J Cardiovasc Imaging*. 2021;37(9):2707–2716. doi:[10.1007/s10554-021-02235-x](https://doi.org/10.1007/s10554-021-02235-x). <https://www.ncbi.nlm.nih.gov/pubmed/33837864>
- Tan TS, Turan Serifler N, Demirtola AI, et al. **Invasive validation of the left ventricular global longitudinal strain for estimating left ventricular filling pressure.** *Echocardiography*. 2021;38(7):1133–1140. doi:[10.1111/echo.15127](https://doi.org/10.1111/echo.15127). <https://www.ncbi.nlm.nih.gov/pubmed/34114248>
- Tanacli R, Hashemi D, Neye M, et al. **Multilayer myocardial strain improves the diagnosis of heart failure with preserved ejection fraction.** *ESC Heart Fail*. 2020;7(5):3240–3245. doi:[10.1002/ehf2.12826](https://doi.org/10.1002/ehf2.12826). <https://www.ncbi.nlm.nih.gov/pubmed/32567247>
- Telles F, Nanayakkara S, Evans S, et al. **Impaired left atrial strain predicts abnormal exercise haemodynamics in heart failure with preserved ejection fraction.** *Eur J Heart Fail*. 2019;21(4):495–505. doi:[10.1002/ejhf.1399](https://doi.org/10.1002/ejhf.1399). <https://www.ncbi.nlm.nih.gov/pubmed/30652393>
- Trippel TD, Mende M, Düngen H-D, et al. **The diagnostic and prognostic value of galectin-3 in patients at risk for heart failure with preserved ejection fraction: results from the DIAST-CHF study.** *ESC Heart Fail*. 2021;8(2):829–841. doi:[10.1002/ehf2.13174](https://doi.org/10.1002/ehf2.13174). <https://www.ncbi.nlm.nih.gov/pubmed/33566456>
- Uijl A, Savarese G, Vaartjes I, et al. **Identification of distinct phenotypic clusters in heart failure with preserved ejection fraction**. *Eur J Heart Fail*. 2021;23(6):973–982. doi:[10.1002/ejhf.2169](https://doi.org/10.1002/ejhf.2169). <https://www.ncbi.nlm.nih.gov/pubmed/33779119>
- Unurjargal T, Khorloo C, Ulziisaikhan G, Sodovsuren N, Khasag A. **Screening for asymptomatic heart failure with preserved ejection fraction in mongolian population at high risk.** *Kardiologiia*. 2020;60(4):86–90. doi:[10.18087/cardio.2020.4.n788](https://doi.org/10.18087/cardio.2020.4.n788). <https://www.ncbi.nlm.nih.gov/pubmed/32394862>
- Vaishnav J, Sharma K. **A Stepwise Guide to the Diagnosis and Treatment of Heart Failure With Preserved Ejection Fraction.** *J Card Fail*. 2022;28(6):1016–1030. doi:[10.1016/j.cardfail.2021.12.013](https://doi.org/10.1016/j.cardfail.2021.12.013). <https://www.ncbi.nlm.nih.gov/pubmed/34968656>
- van de Bovenkamp AA, Wijkstra N, Oosterveer FPT, et al. **The Value of Passive Leg Raise During Right Heart Catheterization in Diagnosing Heart Failure With Preserved Ejection Fraction.** *Circ Heart Fail*. 2022;15(4):e008935. doi:[10.1161/CIRCHEARTFAILURE.121.008935](https://doi.org/10.1161/CIRCHEARTFAILURE.121.008935). <https://www.ncbi.nlm.nih.gov/pubmed/35311526>
- Verbrugge FH, Omote K, Reddy YNV, Sorimachi H, Obokata M, Borlaug BA. **Heart failure with preserved ejection fraction in patients with normal natriuretic peptide levels is associated with increased morbidity and mortality**. *Eur Heart J*. 2022;43(20):1941–1951. doi:[10.1093/eurheartj/ehab911](https://doi.org/10.1093/eurheartj/ehab911). <https://www.ncbi.nlm.nih.gov/pubmed/35139159>
- Verbrugge FH, Reddy YNV, Sorimachi H, Omote K, Carter RE, Borlaug BA. **Diagnostic scores predict morbidity and mortality in patients hospitalized for heart failure with preserved ejection fraction**. *Eur J Heart Fail*. 2021;23(6):954–963. doi:[10.1002/ejhf.2142](https://doi.org/10.1002/ejhf.2142). <https://www.ncbi.nlm.nih.gov/pubmed/33634544>
- Verwerft J, Verbrugge FH, Claessen G, Herbots L, Dendale P, Gevaert AB. **Exercise Systolic Reserve and Exercise Pulmonary Hypertension Improve Diagnosis of Heart Failure With Preserved Ejection Fraction.** *Front Cardiovasc Med*. 2022;9:814601. doi:[10.3389/fcvm.2022.814601](https://doi.org/10.3389/fcvm.2022.814601). <https://www.ncbi.nlm.nih.gov/pubmed/35224049>
- Ward M, Yeganegi A, Baicu CF, et al. **Ensemble machine learning model identifies patients with HFpEF from matrix-related plasma biomarkers.** *Am J Physiol Heart Circ Physiol*. 2022;322(5):H798–H805. doi:[10.1152/ajpheart.00497.2021](https://doi.org/10.1152/ajpheart.00497.2021). <https://www.ncbi.nlm.nih.gov/pubmed/35275763>
- Wong LL, Zou R, Zhou L, et al. **Combining Circulating MicroRNA and NT-proBNP to Detect and Categorize Heart Failure Subtypes.** *J Am Coll Cardiol*. 2019;73(11):1300–1313. doi:[10.1016/j.jacc.2018.11.060](https://doi.org/10.1016/j.jacc.2018.11.060). <https://www.ncbi.nlm.nih.gov/pubmed/30898206>
- Yang F, Wang Q, Zhi G, et al. **The application of lung ultrasound in acute decompensated heart failure in heart failure with preserved and reduced ejection fraction.** *Echocardiography*. 2017;34(10):1462–1469. doi:[10.1111/echo.13646](https://doi.org/10.1111/echo.13646). <https://www.ncbi.nlm.nih.gov/pubmed/28980408>
- Yang J.F., Hua Wang H., Chai K.E., Li Y.Y., Fang F.A.N.G. **Analysis of pathological features in elderly patients with heart failure with preserved ejection fraction**. *Eur J Heart Fail*. 2017;19(Supplement 1):326–327. doi:[10.1002/ejhf.833](https://doi.org/10.1002/ejhf.833).
- Ye Z, Miranda WR, Yeung DF, Kane GC, Oh JK. **Left Atrial Strain in Evaluation of Heart Failure with Preserved Ejection Fraction.** *J Am Soc Echocardiogr*. 2020;33(12):1490–1499. doi:[10.1016/j.echo.2020.07.020](https://doi.org/10.1016/j.echo.2020.07.020). <https://www.ncbi.nlm.nih.gov/pubmed/32981787>
- Zambelli G., Senni M., Gori M., Ghirardi A., Gavazzi A., Iacovoni A. **Echocardiographic phenotypes of acute hfpef: A pilot study**. *Eur Heart J Suppl*. 2020;22(SUPPL G):G9. doi:[10.1093/eurheartj/suaa105](https://doi.org/10.1093/eurheartj/suaa105).
- Zhang S, Zhou Y, Ma Y, Li Z, Hou Y. **The diagnostic value of peroxisome proliferator-activated receptor-γ coactivator-1α in identifying different chronic heart failure phenotypes.** *Front Cardiovasc Med*. 2022;9:973705. doi:[10.3389/fcvm.2022.973705](https://doi.org/10.3389/fcvm.2022.973705). <https://www.ncbi.nlm.nih.gov/pubmed/36148072>
- Zou H, Zhao X, Ce X, et al. **Characterization of patient-specific biventricular mechanics in heart failure with preserved ejection fraction: Hyperelastic warping.** *Annu Int Conf IEEE Eng Med Biol Soc*. 2016;2016:4149–4152. doi:[10.1109/EMBC.2016.7591640](https://doi.org/10.1109/EMBC.2016.7591640).
- Cohen-Solal A, Philip J-L, Picard F, et al. **Iron deficiency in heart failure patients: the French CARENFER prospective study.** *ESC Heart Fail*. 2022;9(2):874–884. doi:[10.1002/ehf2.13850](https://doi.org/10.1002/ehf2.13850). <https://www.ncbi.nlm.nih.gov/pubmed/35170249>
- Elio Gorga E., Sciatti E., Regazzoni V., et al. **Heart failure with preserved ejection fraction: Is the role of arterial stiffness established?** *Eur J Heart Fail*. 2016;18(SUPPL. 1):421–422. doi:[10.1002/ejhf.539](https://doi.org/10.1002/ejhf.539).
- Imran TF, Kurgansky KE, Patel YR, et al. **Serial sodium values and adverse outcomes in heart failure with preserved ejection fraction.** *Int J Cardiol*. 2019;290:119–124. doi:[10.1016/j.ijcard.2019.03.040](https://doi.org/10.1016/j.ijcard.2019.03.040). <https://www.ncbi.nlm.nih.gov/pubmed/30929975>
- McDonagh TA, Metra M, Adamo M, et al. **2021 ESC Guidelines for the diagnosis and treatment of acute and chronic heart failure**. *Eur Heart J*. 2021;42(36):3599-3726. doi: 10.1093/eurheartj/ehab368.
- Bozkurt B, Coats AJS, Tsutsui H, et al. **Universal definition and classification of heart failure: a report of the Heart Failure Society of America, Heart Failure Association of the European Society of Cardiology, Japanese Heart Failure Society and Writing Committee of the Universal Definition of Heart Failure: Endorsed by the Canadian Heart Failure Society, Heart Failure Association of India, Cardiac Society of Australia and New Zealand, and Chinese Heart Failure Association**. *Eur J Heart Fail*. 2021;23(3):352-380. doi: 10.1002/ejhf.2115.
- Mueller C, McDonald K, de Boer RA, et al. **Heart Failure Association of the European Society of Cardiology practical guidance on the use of natriuretic peptide concentrations**. *Eur J Heart Fail.* 2019;21:715-731. doi: 10.1002/ejhf.1494.
- Miñana G, de la Espriella R, Palau Pet al. **Carbohydrate antigen 125 and risk of heart failure readmissions in patients with heart failure and preserved ejection fraction**. *Sci Rep*. 2022;12(1):1344. doi: 10.1038/s41598-022-05328-2.
- Donal E, Galli E, Fraser AG. **Non-invasive estimation of left heart filling pressures: another nail in the coffin for E/e’?** *Eur J Heart Fail* 2017;19:1661–1663. doi: 10.1002/ejhf.944.
- Choi KH, Yang JH, Seo JH, Hong D, Youn T, Joh HS, et al. **Discriminative Role of Invasive Left Heart Catheterization in Patients Suspected of Heart Failure With Preserved Ejection Fraction**. *J Am Heart Assoc*. 2023:e027581. doi: 10.1161/JAHA.122.027581.
- Oh JK, Miranda WR, Kane GC. **Diagnosis of Heart Failure With Preserved Ejection Fraction Relies on Detection of Increased Diastolic Filling Pressure, But How?** *J Am Heart Assoc*. 2023:e028867. doi: 10.1161/JAHA.122.028867.
- Anker SD, Butler J, Filippatos G, Ferreira JP, Bocchi E, Böhm M, et al. **Empagliflozin in Heart Failure with a Preserved Ejection Fraction**. *N Engl J Med*. 2021;385(16):1451-1461. doi: 10.1056/NEJMoa2107038.
- Solomon SD, McMurray JJV, Claggett B, de Boer RA, DeMets D, Hernandez AF, et al. **Dapagliflozin in Heart Failure with Mildly Reduced or Preserved Ejection Fraction**. *N Engl J Med*. 2022;387(12):1089-1098. doi: 10.1056/NEJMoa2206286.
- Solomon SD, McMurray JJV, Anand Is, et al. **Angiotensin-Neprilysin Inhibition in Heart Failure with Preserved Ejection Fraction**. *N Engl J Med*. 2019;381(17):1609-1620. doi: 10.1056/NEJMoa1908655.

**2.3. Amyloidosis**

- Tomasoni D, Aimo A, Merlo M, et al. **Value of the HFA-PEFF and H2 FPEF scores in patients with heart failure and preserved ejection fraction caused by cardiac amyloidosis**. *Eur J Heart Fail*. 2022;24(12):2374–2386. doi:[10.1002/ejhf.2616](https://doi.org/10.1002/ejhf.2616). <https://www.ncbi.nlm.nih.gov/pubmed/35855616>
- Aimo A, Merlo M, Porcari A, et al. **Redefining the epidemiology of cardiac amyloidosis. A systematic review and meta-analysis of screening studies.** *Eur J Heart Fail*. 2022. doi:[10.1002/ejhf.2532](https://doi.org/10.1002/ejhf.2532). <https://www.ncbi.nlm.nih.gov/pubmed/35509173>
- Bennani Smires Y, Victor G, Ribes D, et al. **Pilot study for left ventricular imaging phenotype of patients over 65 years old with heart failure and preserved ejection fraction: the high prevalence of  amyloid cardiomyopathy.** *Int J Cardiovasc Imaging*. 2016;32(9):1403–1413. doi:[10.1007/s10554-016-0915-z](https://doi.org/10.1007/s10554-016-0915-z). <https://www.ncbi.nlm.nih.gov/pubmed/27240600>
- Chen W, Ton V-K, Dilsizian V. **Clinical Phenotyping of Transthyretin Cardiac Amyloidosis with Bone-Seeking Radiotracers in Heart Failure with Preserved Ejection Fraction.** *Curr Cardiol Rep*. 2018;20(4):23. doi:[10.1007/s11886-018-0970-2](https://doi.org/10.1007/s11886-018-0970-2). <https://www.ncbi.nlm.nih.gov/pubmed/29520480>
- de Marneffe N, Dulgheru R, Ancion A, Moonen M, Lancellotti P. **Cardiac amyloidosis: a review of the literature.** *Acta Cardiol*. 2022;77(8):683–692. doi:[10.1080/00015385.2021.1992990](https://doi.org/10.1080/00015385.2021.1992990). <https://www.ncbi.nlm.nih.gov/pubmed/35852493>
- Di Giovanni B, Gustafson D, Delgado DH. **Amyloid transthyretin cardiac amyloidosis: diagnosis and management.** *Expert Rev Cardiovasc Ther*. 2019;17(9):673–681. doi:[10.1080/14779072.2019.1662723](https://doi.org/10.1080/14779072.2019.1662723). <https://www.ncbi.nlm.nih.gov/pubmed/31478389>
- Gertz MA, Dispenzieri A. **Systemic Amyloidosis Recognition, Prognosis, and Therapy: A Systematic Review.** *JAMA*. 2020;324(1):79–89. doi:[10.1001/jama.2020.5493](https://doi.org/10.1001/jama.2020.5493). <https://www.ncbi.nlm.nih.gov/pubmed/32633805>
- Hasib Sidiqi M, Gertz MA. **Immunoglobulin light chain amyloidosis diagnosis and treatment algorithm 2021.** *Blood Cancer J*. 2021;11(5):90. doi:[10.1038/s41408-021-00483-7](https://doi.org/10.1038/s41408-021-00483-7). <https://www.ncbi.nlm.nih.gov/pubmed/33993188>
- Huang Y-H, Lin Y-H, Yen R-F, et al. **2021 Advocacy Statements for the Role of (99m)Tc-Pyrophosphate Scintigraphy in the Diagnosis of Transthyretin Cardiac Amyloidosis: A Report of the Taiwan  Society of Cardiology and the Society of Nuclear Medicine of the Republic of  China.** *Acta Cardiol Sin*. 2021;37(3):221–231. doi:[10.6515/ACS.202105_37(3).20210420A](https://doi.org/10.6515/ACS.202105_37(3).20210420A). <https://www.ncbi.nlm.nih.gov/pubmed/33976505>
- Ihne S, Morbach C, Obici L, Palladini G, Störk S. **Amyloidosis in Heart Failure.** *Curr Heart Fail Rep*. 2019;16(6):285–303. doi:[10.1007/s11897-019-00446-x](https://doi.org/10.1007/s11897-019-00446-x). <https://www.ncbi.nlm.nih.gov/pubmed/31782077>
- Inomata T, Tahara N, Nakamura K, et al. **Diagnosis of wild-type transthyretin amyloid cardiomyopathy in Japan: red-flag symptom clusters and diagnostic algorithm.** *ESC Heart Fail*. 2021;8(4):2647–2659. doi:[10.1002/ehf2.13473](https://doi.org/10.1002/ehf2.13473). <https://www.ncbi.nlm.nih.gov/pubmed/34137515>
- Mereles D., Aurich M., Greiner S., et al. **Diagnosis of cardiac involvement in systemic amyloidosis by state-of-the-art echocardiography: where are we now?** *Expert Opin Orphan Drugs*. 2016;4(6):639–648. doi:[10.1080/21678707.2016.1179573](https://doi.org/10.1080/21678707.2016.1179573).
- Migliaccio M.G., Iodice F., Di Mauro M., et al. **Cardiac amyloidosis: Diagnostic tools for a challenging disease**. *Cardiogenetics*. 2021;11(3):111–121. doi:[10.3390/CARDIOGENETICS11030012](https://doi.org/10.3390/CARDIOGENETICS11030012).
- Morgenstern R., Pozniakoff T., Castano A., et al. **Pyp imaging with czt cameras is a sensitive and specific imaging modality to diagnose TTR cardiac amyloidosis**. *J Am Coll Cardiol*. 2018;71(11 Supplement 1). doi:[10.1016/S0735-1097%2818%2932235-6](https://doi.org/10.1016/S0735-1097%2818%2932235-6).
- Ochi Y, Kubo T, Baba Y, et al. **Wild-Type Transthyretin Amyloidosis in Female Patients　- Consideration of Sex Differences.** *Circ Rep*. 2021;3(8):465–471. doi:[10.1253/circrep.CR-21-0067](https://doi.org/10.1253/circrep.CR-21-0067). <https://www.ncbi.nlm.nih.gov/pubmed/34414336>
- Rapezzi C, Vergaro G, Emdin M, et al. **The revolution of ATTR amyloidosis in cardiology: certainties, gray zones and perspectives.** *Minerva Cardiol Angiol*. 2022;70(2):248–257. doi:[10.23736/S2724-5683.21.05926-3](https://doi.org/10.23736/S2724-5683.21.05926-3). <https://www.ncbi.nlm.nih.gov/pubmed/35412035>
- Rezk T., Fontana M., Gillmore J.D. **A review of the criteria for non-invasive diagnosis of cardiac transthyretin amyloidosis**. *Expert Opin Orphan Drugs*. 2021;9(3):87–94. doi:[10.1080/21678707.2021.1898371](https://doi.org/10.1080/21678707.2021.1898371).
- Sennott J, Ananthasubramaniam K. **Multimodality imaging approach to cardiac amyloidosis: part 2.** *Heart Fail Rev*. 2022;27(5):1515–1530. doi:[10.1007/s10741-021-10179-6](https://doi.org/10.1007/s10741-021-10179-6). <https://www.ncbi.nlm.nih.gov/pubmed/34694574>
- Shah K., Saifuddin A., Kempf D., Yudd A.P., Kempf J.S. **Enlarged atria in the right hemithorax and heart to contralateral ratio: A 99mTc-PYP imaging potential pitfall in the evaluation of transthyretin cardiac amyloidosis**. *Clin Nucl Med*. 2020;45(4):295. doi:[10.1097/RLU.0000000000002990](https://doi.org/10.1097/RLU.0000000000002990).
- Sperry BW, Gonzalez MH, Brunken R, Cerqueira MD, Hanna M, Jaber WA. **Non-cardiac uptake of technetium-99m pyrophosphate in transthyretin cardiac amyloidosis.** *J Nucl Cardiol*. 2019;26(5):1630–1637. doi:[10.1007/s12350-017-1166-7](https://doi.org/10.1007/s12350-017-1166-7). <https://www.ncbi.nlm.nih.gov/pubmed/29344917>
- Sperry BW, Vadalia A. **Primer on the Differential Diagnosis and Workup for Transthyretin Cardiac Amyloidosis.** *Am J Cardiol*. 2022;185 Suppl 1:S11–S16. doi:[10.1016/j.amjcard.2022.10.052](https://doi.org/10.1016/j.amjcard.2022.10.052). <https://www.ncbi.nlm.nih.gov/pubmed/36549787>
- Tini G, Sessarego E, Benenati S, et al. **Yield of bone scintigraphy screening for transthyretin-related cardiac amyloidosis in different conditions: Methodological issues and clinical  implications.** *Eur J Clin Invest*. 2021;51(12):e13665. doi:[10.1111/eci.13665](https://doi.org/10.1111/eci.13665). <https://www.ncbi.nlm.nih.gov/pubmed/34390490>
- Yamamoto H, Yokochi T. **Transthyretin cardiac amyloidosis: an update on diagnosis and treatment.** *ESC Heart Fail*. 2019;6(6):1128–1139. doi:[10.1002/ehf2.12518](https://doi.org/10.1002/ehf2.12518). <https://www.ncbi.nlm.nih.gov/pubmed/31553132>
- Yang H, Li R, Ma F, et al. **An echo score raises the suspicion of cardiac amyloidosis in Chinese with heart failure with preserved ejection fraction.** *ESC Heart Fail*. 2022;9(6):4280–4290. doi:[10.1002/ehf2.14164](https://doi.org/10.1002/ehf2.14164). <https://www.ncbi.nlm.nih.gov/pubmed/36128643>

**2.4. Discussion.**

- Çavuşoğlu Y, Çelik A, Altay H, et al. **Heart failure with non-reduced ejection fraction: Epidemiology, pathophysiology, phenotypes, diagnosis and treatment approaches.** *Turk Kardiyol Dern Ars*. 2022;50(Supp1):S1–S34. doi:[10.5543/tkda.2022.S1](https://doi.org/10.5543/tkda.2022.S1). <https://www.ncbi.nlm.nih.gov/pubmed/35969235>
- Gevaert AB, Kataria R, Zannad F, et al. **Heart failure with preserved ejection fraction: recent concepts in diagnosis, mechanisms and management**. *Heart*. 2022;108(17):1342–1350. doi:[10.1136/heartjnl-2021-319605](https://doi.org/10.1136/heartjnl-2021-319605). <https://www.ncbi.nlm.nih.gov/pubmed/35022210>
- Hamdani N, Costantino S, Mügge A, et al. **Leveraging clinical epigenetics in heart failure with preserved ejection fraction: a call for individualized therapies.** *Eur Heart J*. 2021;42(20):1940–1958. doi:[10.1093/eurheartj/ehab197](https://doi.org/10.1093/eurheartj/ehab197). <https://www.ncbi.nlm.nih.gov/pubmed/36282124>
- Hashemi D, Mende M, Trippel TD, et al. **Evaluation of the HFA-PEFF Score: results from the prospective DIAST-CHF cohort.** *ESC Heart Fail*. 2022;9(6):4120–4128. doi:[10.1002/ehf2.14131](https://doi.org/10.1002/ehf2.14131). <https://www.ncbi.nlm.nih.gov/pubmed/36070881>
- Luo H, Xu Y, Yue F, Zhang C, Chen C. **Quality of inclusion criteria in the registered clinical trials of heart failure with preserved ejection fraction: Is it time for a change?** *Int J Cardiol*. 2018;254:210–214. doi:[10.1016/j.ijcard.2017.12.025](https://doi.org/10.1016/j.ijcard.2017.12.025). <https://www.ncbi.nlm.nih.gov/pubmed/29254883>
- Méndez AB, Azancot MA, Olivella A, Soler MJ. **New aspects in cardiorenal syndrome and HFpEF.** *Clin Kidney J*. 2022;15(10):1807–1815. doi:[10.1093/ckj/sfac133](https://doi.org/10.1093/ckj/sfac133). <https://www.ncbi.nlm.nih.gov/pubmed/36158149>
- Wintrich J, Abdin A, Böhm M. **Management strategies in heart failure with preserved ejection fraction.** *Herz*. 2022;47(4):332–339. doi:[10.1007/s00059-022-05119-5](https://doi.org/10.1007/s00059-022-05119-5). <https://www.ncbi.nlm.nih.gov/pubmed/35524007>

**2.5. Excluded.**

- Abdellatif M, Leite S, Alaa M, et al. **Spectral transfer function analysis of respiratory hemodynamic fluctuations predicts end-diastolic stiffness in preserved ejection fraction heart failure.** *Am J Physiol Heart Circ Physiol*. 2016;310(1):H4-13. doi:[10.1152/ajpheart.00399.2015](https://doi.org/10.1152/ajpheart.00399.2015). <https://www.ncbi.nlm.nih.gov/pubmed/26475584>
- Boutagy NE, Feher A, Alkhalil I, Umoh N, Sinusas AJ. **Molecular Imaging of the Heart.** *Compr Physiol*. 2019;9(2):477–533. doi:[10.1002/cphy.c180007](https://doi.org/10.1002/cphy.c180007). <https://www.ncbi.nlm.nih.gov/pubmed/30873600>
- Chou R.-H., Hsu C.-Y., Huang P.-H., Chen J.-W., Lin S.-J. **Circulating fibroblast growth factor 21 is associated with diastolic dysfunction in patients with preserved ejection fraction**. *Eur Heart J*. 2016;37(Supplement 1):441–442. doi:[10.1093/eurheartj/ehw432](https://doi.org/10.1093/eurheartj/ehw432).
- Fabiani I, Pugliese NR, La Carrubba S, et al. **Interactive role of diastolic dysfunction and ventricular remodeling in asymptomatic subjects at increased risk of heart failure.** *Int J Cardiovasc Imaging*. 2019;35(7):1231–1240. doi:[10.1007/s10554-019-01560-6](https://doi.org/10.1007/s10554-019-01560-6). <https://www.ncbi.nlm.nih.gov/pubmed/30815808>
- Ghionzoli N, Gismondi A, Mandoli GE, et al. **Left Ventricular Mass with Delayed Enhancement as a Predictor of Major Events in Patients with Myocarditis with Preserved Ejection Fraction.** *J Clin Med*. 2022;11(20). doi:[10.3390/jcm11206082](https://doi.org/10.3390/jcm11206082). <https://www.ncbi.nlm.nih.gov/pubmed/36294403>
- He T, Mischak M, Clark AL, et al. **Urinary peptides in heart failure: a link to molecular pathophysiology.** *Eur J Heart Fail*. 2021;23(11):1875–1887. doi:[10.1002/ejhf.2195](https://doi.org/10.1002/ejhf.2195). <https://www.ncbi.nlm.nih.gov/pubmed/33881206>
- Hunter WG, Kelly JP, McGarrah RW 3rd, et al. **Metabolomic Profiling Identifies Novel Circulating Biomarkers of Mitochondrial Dysfunction Differentially Elevated in Heart Failure With Preserved Versus  Reduced Ejection Fraction: Evidence for Shared Metabolic Impairments in Clinical  Heart Failure.** *J Am Heart Assoc*. 2016;5(8). doi:[10.1161/JAHA.115.003190](https://doi.org/10.1161/JAHA.115.003190). <https://www.ncbi.nlm.nih.gov/pubmed/27473038>
- Karakurt A., Yildiz C., Yildiz A., et al. **Early detection strain/strain rate and time to strain/strain rate abnormalities for left atrial mechanical function in hypertensive patients**. *Acta Cardiol*. 2019;74(2):141–151. doi:[10.1080/00015385.2018.1475031](https://doi.org/10.1080/00015385.2018.1475031).
- Li JK-J, Kaya M, Kerkhof PLM. **Quantitative cardiology and computer modeling analysis of heart failure in systole and in diastole.** *Comput Biol Med*. 2018;103:252–261. doi:[10.1016/j.compbiomed.2018.10.030](https://doi.org/10.1016/j.compbiomed.2018.10.030). <https://www.ncbi.nlm.nih.gov/pubmed/30396041>
- Santoro C, Sorrentino R, Esposito R, et al. **Cardiopulmonary exercise testing and echocardiographic exam: an useful interaction.** *Cardiovasc Ultrasound*. 2019;17(1):29. doi:[10.1186/s12947-019-0180-0](https://doi.org/10.1186/s12947-019-0180-0). <https://www.ncbi.nlm.nih.gov/pubmed/31796047>
- Tisdale R., Heidenreich P. **Applying natural language processing of echocardiography reports to describe epidemiology of heart failure**. *J Am Coll Cardiol*. 2018;71(11 Supplement 1). doi:[10.1016/S0735-1097%2818%2931253-1](https://doi.org/10.1016/S0735-1097%2818%2931253-1).
- Zhu Y, Jackson D, Hunter B, et al. **Models of cardiovascular surgery biobanking to facilitate translational research and precision medicine.** *ESC Heart Fail*. 2022;9(1):21–30. doi:[10.1002/ehf2.13768](https://doi.org/10.1002/ehf2.13768). <https://www.ncbi.nlm.nih.gov/pubmed/34931483>

**Supplementary figure 1. Study flow diagram**


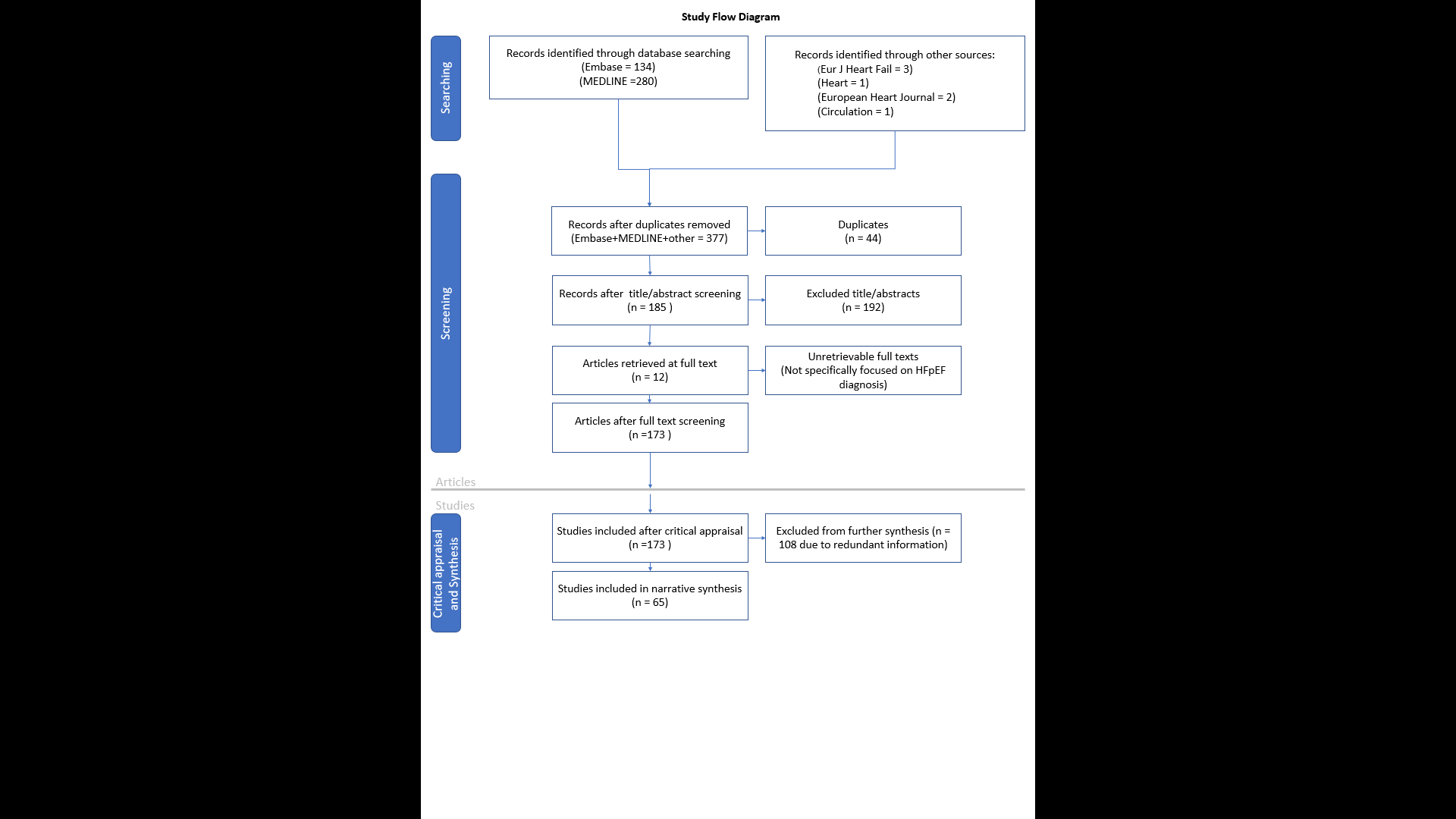

Supplement: Supplementary file 1 — Supplementary file1 (DOCX 135 KB) [file 10741_2023_10360_MOESM1_ESM.docx]
